# Radiomics for the detection of diffusely impaired myocardial perfusion: A proof-of-concept study using 13N-ammonia positron emission tomography

**DOI:** 10.1007/s12350-022-03179-y

**Published:** 2023-01-05

**Authors:** Ganna Degtiarova, Chrysoula Garefa, Reto Boehm, Domenico Ciancone, Daniel Sepulcri, Catherine Gebhard, Andreas A. Giannopoulos, Aju P. Pazhenkottil, Philipp A. Kaufmann, Ronny R. Buechel

**Affiliations:** Department of Nuclear Medicine, Cardiac Imaging, University and University Hospital Zurich, Ramistrasse 100, 8091 Zurich, Switzerland

**Keywords:** Radiomic features, positron emission tomography, myocardial flow reserve

## Abstract

**Aim:**

The current proof-of-concept study investigates the value of radiomic features from normal 13N-ammonia positron emission tomography (PET) myocardial retention images to identify patients with reduced global myocardial flow reserve (MFR).

**Methods:**

Data from 100 patients with normal retention 13N-ammonia PET scans were divided into two groups, according to global MFR (i.e., < 2 and ≥ 2), as derived from quantitative PET analysis. We extracted radiomic features from retention images at each of five different gray-level (GL) discretization (8, 16, 32, 64, and 128 bins). Outcome independent and dependent feature selection and subsequent univariate and multivariate analyses was performed to identify image features predicting reduced global MFR.

**Results:**

A total of 475 radiomic features were extracted per patient. Outcome independent and dependent feature selection resulted in a remainder of 35 features. Discretization at 16 bins (GL16) yielded the highest number of significant predictors of reduced MFR and was chosen for the final analysis. GLRLM_GLNU was the most robust parameter and at a cut-off of 948 yielded an accuracy, sensitivity, specificity, negative and positive predictive value of 67%, 74%, 58%, 64%, and 69%, respectively, to detect diffusely impaired myocardial perfusion.

**Conclusion:**

A single radiomic feature (GLRLM_GLNU) extracted from visually normal 13N-ammonia PET retention images independently predicts reduced global MFR with moderate accuracy. This concept could potentially be applied to other myocardial perfusion imaging modalities based purely on relative distribution patterns to allow for better detection of diffuse disease.

**Supplementary Information:**

The online version contains supplementary material available at 10.1007/s12350-022-03179-y.

## Introduction

Nuclear myocardial perfusion imaging (MPI) is well established for the assessment of suspected or known coronary artery disease (CAD) and is widely implemented in clinical practice. Single-photon emission computed tomography (SPECT), however, is inherently limited in detecting diffuse myocardial perfusion abnormalities, as this technique relies on normally perfused myocardium as a reference for normalization of myocardial radionuclide retention. This becomes relevant, for example, in a setting of multi-vessel CAD and/or microvascular disease, explaining the moderate sensitivity of SPECT in these clinical situations.^1,2^ Contrary to conventional SPECT, positron emission tomography (PET) imaging allows for absolute quantification of myocardial perfusion, thus overcoming the above-mentioned shortcomings of SPECT.^3,4^ Myocardial blood flow (MBF) and myocardial flow reserve (MFR), assessed with PET, provide a quantitative measure integrating the hemodynamic consequences of focal lesions, diffuse lesions, small vessel disease, and microvascular dysfunction on tissue perfusion.

Despite the obvious advantages of PET MPI, its wide implementation is currently hampered by limited availability due to relatively high up-front costs and the need for an on-site cyclotron or generator due to the short half-life of currently available PET perfusion tracers. On the other hand, while newest-generation SPECT cameras with cadmium-zinc-telluride-base detector technology have been shown to allow for dynamic image acquisition, enabling accurate MBF quantitation,^5–7^ their availability currently remains limited as well. Thus, the majority of cardiac nuclear studies for evaluating CAD is currently performed on conventional SPECT cameras, which do not allow for quantification of MBF and are prone to false negative findings in patients with balanced multi-vessel disease, left main coronary artery disease or microvascular disease, which are associated with an increased cardiovascular risk.^8,9^ It is against this background that substantial efforts have been made to identify imaging features within the SPECT data itself to detect the presence of balanced ischemia or coronary microvascular disease. Various approaches have, however, demonstrated moderate performance at best, not qualifying them as robust and reliable markers in clinical routine for the detection of diffusely impaired myocardial perfusion.^10–12^

By contrast, the concept of radiomics as a novel supportive approach for image analysis in cardiac nuclear imaging is promising in this regard, as it allows assessing a multitude of subtle image features, which are imperceptible to the naked human eye and may potentially be more beneficial than previously addressed markers. Radiomics and machine learning applications in cardiology and cardiovascular imaging are now quite common in research^13^ and a number of studies have already demonstrated a potential clinical value of radiomics in nuclear medicine and radiology, including cardiac CT.^14,15^ However, the literature on its potential value specifically in SPECT MPI remains scarce.

We hypothesize that the application of radiomics to myocardial perfusion radionuclide retention images yields diagnostic value for the detection of diffusely impaired perfusion. The current work aims to provide proof-of-concept using 13N-ammonia PET MPI. Contrary to SPECT MPI, the latter provides the unique advantage of providing both the retention images and the standard of truth through absolute MBF quantification.

## Material and methods

### Patient collection

This is a retrospective single-center matched cohort study including patients with normal retention images on 13N-ammonia PET MPI scans. We identified patients from the Zurich Quantitative PET Registry^16^ with preserved and decreased global MFR as derived from PET quantification, matched by age, gender, and body mass index. Preserved MFR was defined as global MFR ≥ 2 and decreased MFR was defined as global MFR < 2.^17^ This study was approved by the local ethics committee (BASEC-Nr. 2016-09177).

### PET acquisition, reconstruction, and analysis

All patients underwent clinically indicated PET MPI using 13N-ammonia acquired at rest and during pharmacological stress (adenosine infused at 0.14 mg⋅kg^−1^⋅min^−1^ over 6 minutes or single bolus injection of 400 mcg of regadenosone) according to clinical routine. All data were acquired in list-mode on a PET/CT scanner (Discovery DST, Discovery MI or Discovery VCT, all GE Healthcare, Waukesha, WI, USA) as previously reported.^16^ In brief, a body mass index-adapted dose of 13N-ammonia (i.e., 400-1200 Megabecquerels) was injected and the datasets were reconstructed using ordered subset expectation maximum (OSEM, VUE Point HD or VUE Point FX with 2 iterations and 16 subsets), and a 5 mm Hanning filter and standard decay, scatter and sensitivity corrections (voxel size 2.34, 2.34, 2.80-3.27) were applied. Low-dose unenhanced computed tomography was used for attenuation correction. Dynamic datasets were reconstructed from the first 7 minutes of acquisition and consisted of 9 frames of 10 seconds duration, 6 frames of 15 seconds, 3 frames of 20 seconds, 2 frames of 30 seconds and 1 frame of 120 seconds. MBF at rest (corrected for the rate-pressure-product) and during stress and MFR was calculated using commercially available software (QPET 2017.7 Cedars-Sinai Medical Center, Los Angeles, CA, USA). Static datasets were reconstructed from the following 10 minutes of the acquisition.

### Extraction of radiomic features

**Figure 1:**
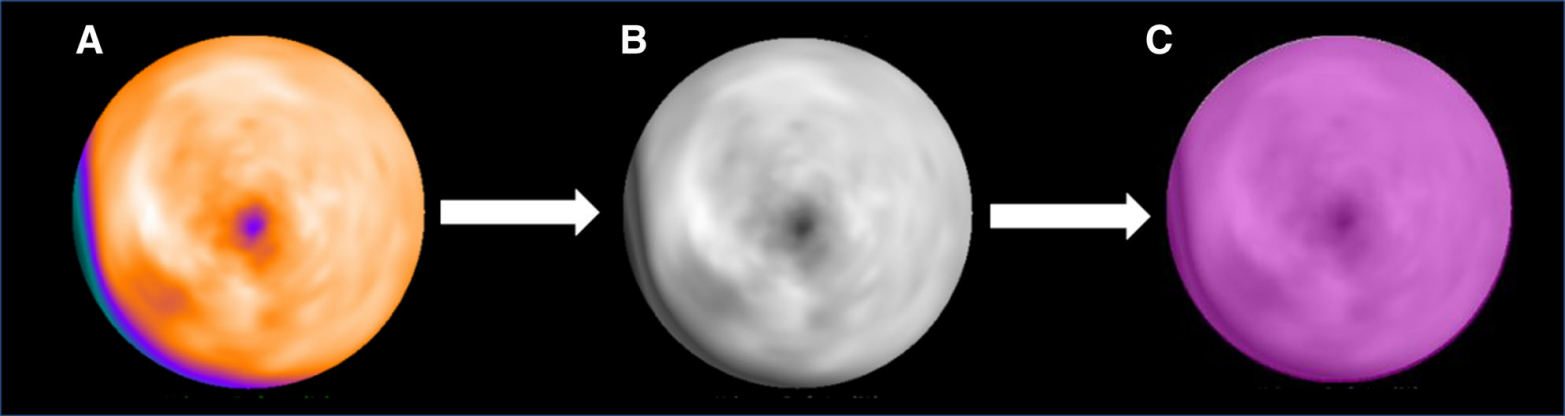
PET image processing for radiomic feature extraction. 13N-NH3 PET stress myocardial perfusion retention images represented as a left ventricular (LV) polar map (**a**) are converted into a grayscale lossless Portable Network Graphic (PNG) file format (**b**). A region of interest (purple) drawn on the whole LV (**c**) in for the subsequent radiomic feature extraction using LifeX software

Polar maps encompassing the left ventricular myocardium were created from the static stress datasets and tracer uptake was normalized to 100% peak activity (Figure 1a). Polar maps were then saved in a lossless Portable Network Graphic (PNG) file format with 256 gray levels (Figure 1b) but with no other post-processing applied. Conversion of the color-coded images into gray levels was performed so as to provide the exact number of levels to the software used in the following steps. Of note, application of a continuous color-scale to the raw SPECT data, whereby brightness of each pixel corresponds to relative radionuclide uptake is a mandatory prerequisite for all further analyses along with the need to not introduce any random compression artifacts. A region of interest was then drawn, encompassing the entire left ventricular polar map (Figure 1c) for the subsequent radiomic feature extraction. Radiomic features were extracted using the Local Image Features Extraction software package (LifeX®, LITO, Orsay, France), validated by the image biomarker standardisation initiative (IBSI).^18^ Two-dimensional settings were used for radiomics analysis. We considered and investigated five different gray-levels (GL) discretization, specifically using 8, 16, 32, 64 and 128 bins. For each GL first- and second-order features (size and shape based–features, image intensity features, voxel relationship features etc.) were computed, resulting in a total of 5 × 95 radiomic features.

### Radiomic feature selection

First, we performed a GL-independent feature selection process, which included all calculated radiomic parameters. We performed a multistep selection procedure to eliminate identical, non-robust, and redundant features in order to reduce the feature-space to a smaller, more meaningful set of parameters that provide a robust characterisation of myocardial 13N-ammonia retention.

#### Outcome independent feature selection

In the first step, we excluded all features that cannot be applied to the nature of our two-dimensional dataset and features whose content did not bring any meaningful information on the GL characterisation.

In the second step the features with low predictive power, that is, with zero or near-zero variance (i.e., var < 0.0025) were excluded. Furthermore, as a large number of features exhibiting similar information may influence the prediction accuracy, we eliminated all features with a pairwise Pearson’s correlation coefficient |ρ|≥ 0.85.

#### Outcome dependent feature selection

From the remaining features, we then identified the most important features using a Boruta algorithm.^19^ In short, the Boruta algorithm performs multiple runs of Random Forest analyses, comparing the relevance of the features to those of the random probes, which are shuffled copies (“shadows”) of the original variables. Variables performing better than the maximum random variable importance were classified as important and were kept for the further analysis, variables performing worse were rejected.

Finally, we performed a GL-dependent univariate logistic regression analysis to investigate which of the remaining radiomic features predict reduced MFR for each of the five acquired GLs and the Benjamini–Hochberg procedure was used to correct for multiple comparisons and to control the false discovery rate (FDR) at 10%.^20^ The GL with the highest number of significant predictors of reduced MFR was selected for the subsequent multivariate logistic regression.

### Statistical analysis

Statistical analysis was performed using SPSS (version 25, IBM Corporation, Armonk, NY, USA) and RStudio (version 1.4, RStudio, Inc., Boston, MA, USA). Normally distributed continuous clinical characteristics parameters of the patients are expressed as mean ± standard deviation, otherwise median and interquartile range are given. Categorical variables are represented as percentages. Unpaired *T*-tests were used for comparison of normally distributed continuous variables. Mann–Whitney *U* test was applied for not normally distributed variables. The correlation between radiomic features was assessed with Pearson correlation coefficients. Univariate und multivariate forward stepwise logistic regression were used to determine the significant predictors of reduced MFR. *P*-values in univariate regression were adjusted for multiple testing with the Benjamini–Hochberg method with an FDR of 10%.^20^ Receiver operating curve (ROC) analysis was applied on continuous features to identify the optimal cut-off values, while area under the curve (AUC) was used to assess the overall model performance. All statistical tests were 2-tailed. A *P*-value of less than 0.05 was considered statistically significant, unless indicated otherwise.

## Results

### Patient characteristics

Fifty patients with decreased MFR (group 1) and 50 patients matched by gender, age and body mass index with preserved MFR (group 2) were randomly selected from the Zurich Quantitative PET Registry, generating a cohort of 100 patients. The patient population consisted of 58 females (58%) and 42 males (42%) with an average age of 64 ± 12 years. Average global MFR in group 1 and group 2 was 1.74 ± 0.18 and 3.20 ± 0.81, respectively (*P* < .0001). A validation cohort of 30 additional patients was randomly selected from the same registry. Of note, data from this validation cohort were not used for feature selection and/or statistical analyses but exclusively to test the final model.

### Radiomic feature selection

**Figure 2:**
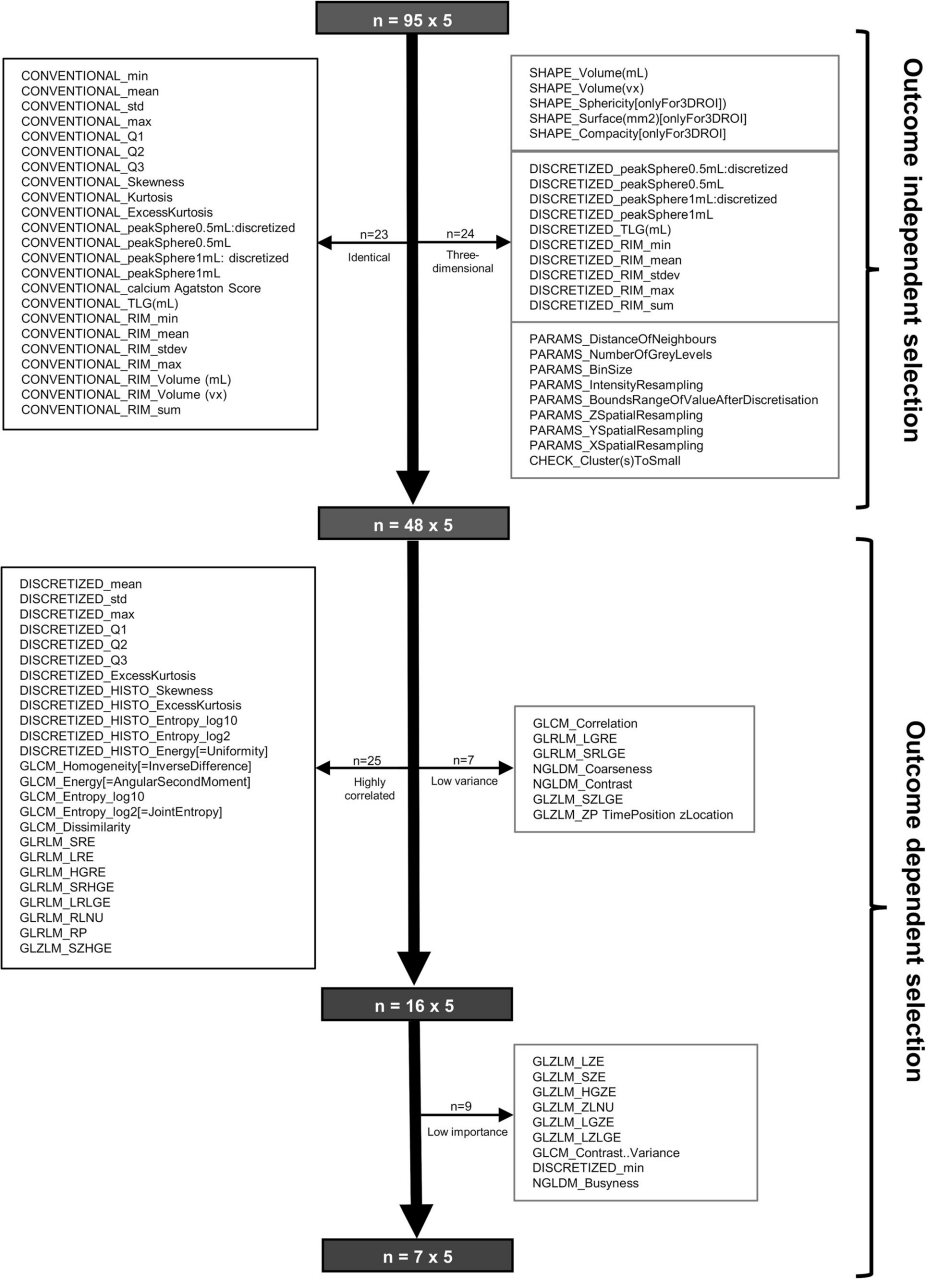
Flow chart of the feature selection algorithm

The detailed overview of feature selection is represented in Figure 2.

#### Outcome independent selection

**Figure 3:**
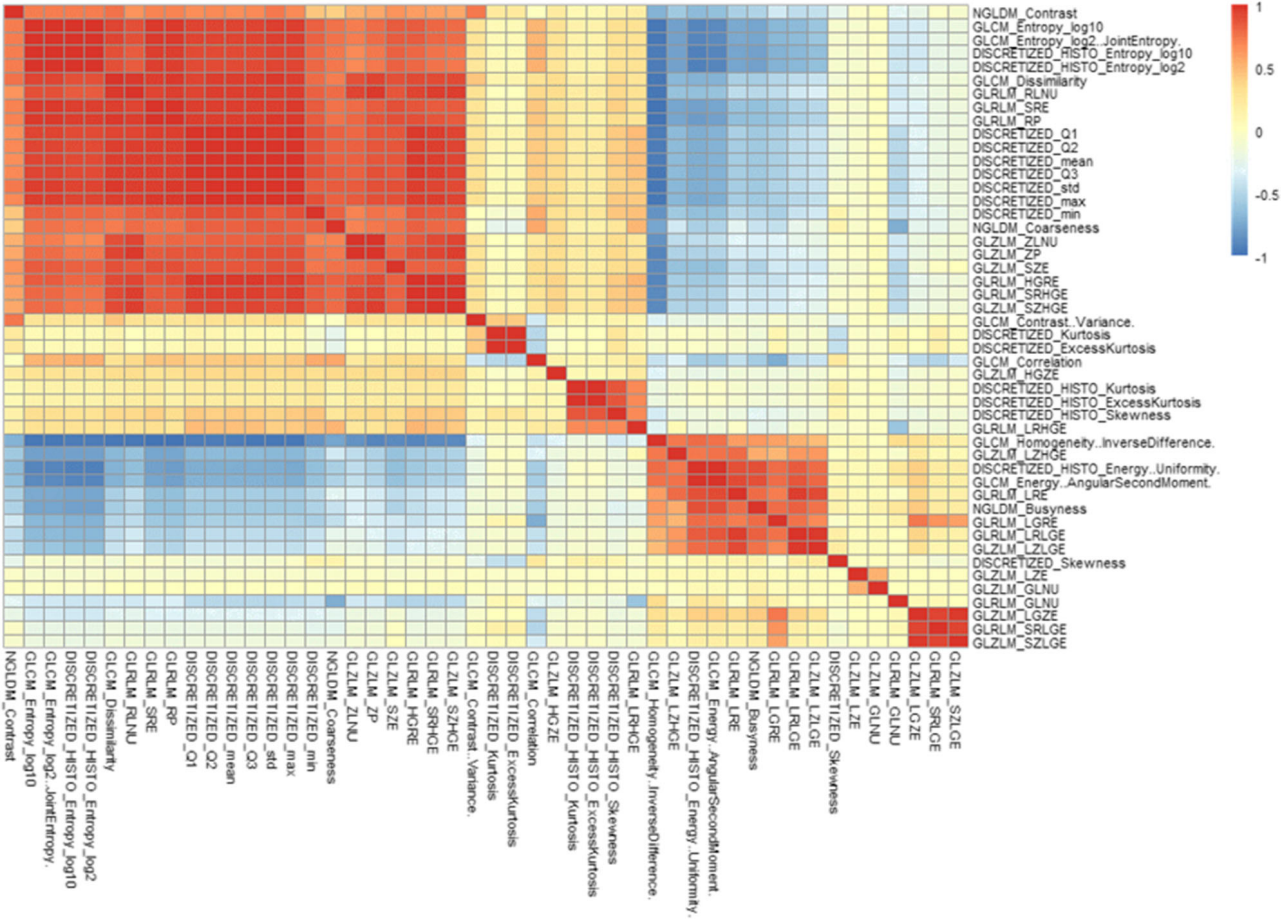
Heatmap of pairwise Pearson’s correlation between radiomic features

Out of 95 radiomic features extracted per each GL, 24 seconds-order statistics features were applicable only to three-dimensional volumes of interest and were removed from our selection. From the remaining 71 features, 23 first-order statistics (conventional) features with identical values across all GLs were removed, given that these features did not provide any additional information on GL characterization. At the next step, 7 features with low variance were revealed and were removed from our selection, leaving 41 remaining features per GL. Afterwards, focusing on the correlation matrix between the radiomic features, we excluded 25 highly correlated features (Figure 3). The outcome independent radiomic feature selection resulted in a remainder of 16 features per GL.

#### Outcome dependent selection

**Figure 4:**
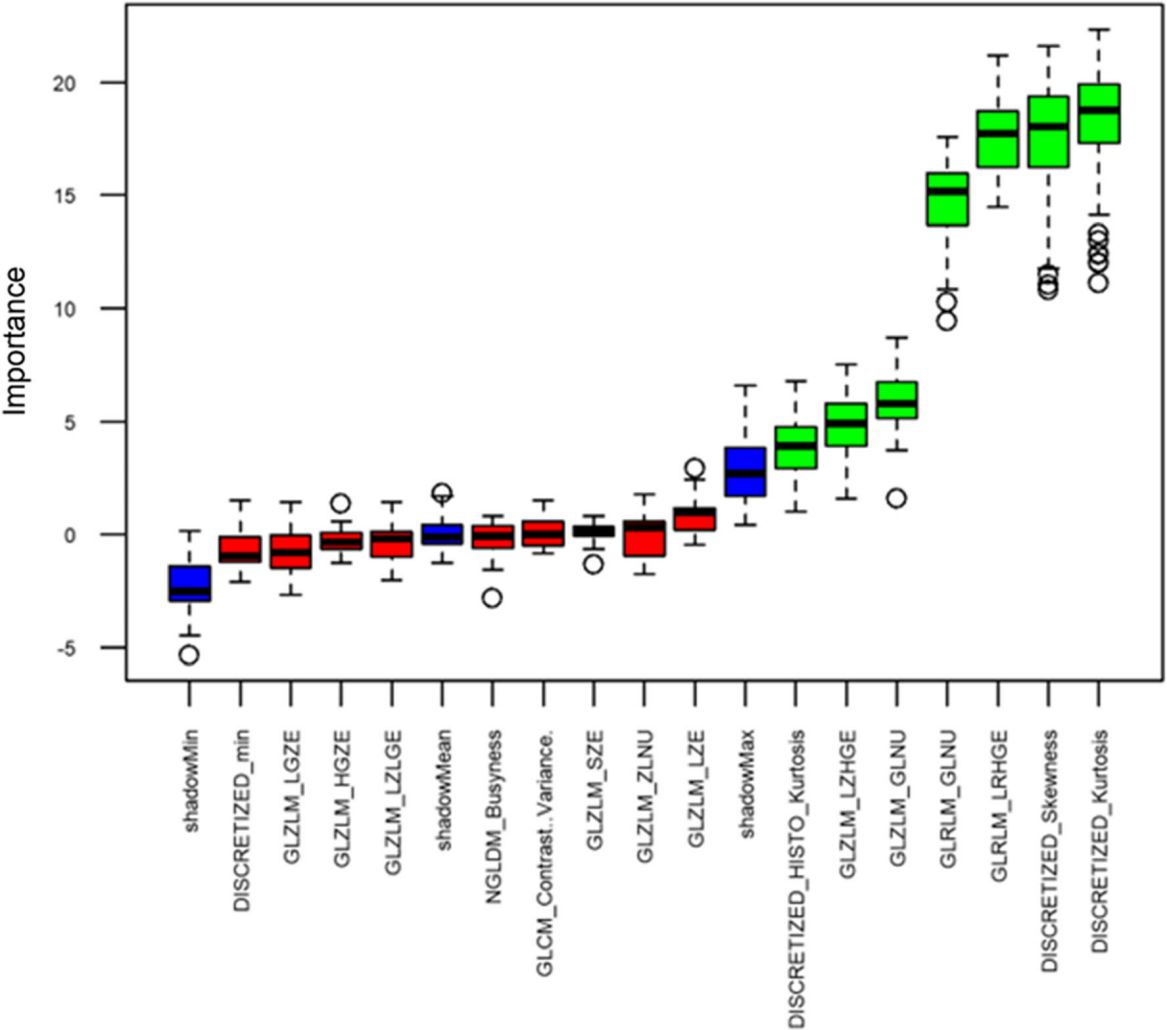
Feature selection with Boruta algorithm. Blue boxes correspond to shadow attributes, green color indicates important attributes, and red boxes indicate attributes that are deemed unimportant

The results of Boruta algorithm (Figure 4) demonstrated that only 7 out of the remaining 16 radiomic features (Discretized_Kurtosis, Discretized_Skewness, GLRLM_LRHGE, GLRLM_GLNU, GLZLM_GLNU, GLZLM_LZHGE, DISCRETIZED_HISTO_Kurtosis) yielded a sufficiently high predictive power for differentiating patients with preserved versus decreased global MFR.

#### Gray level selection

**Figure 5:**
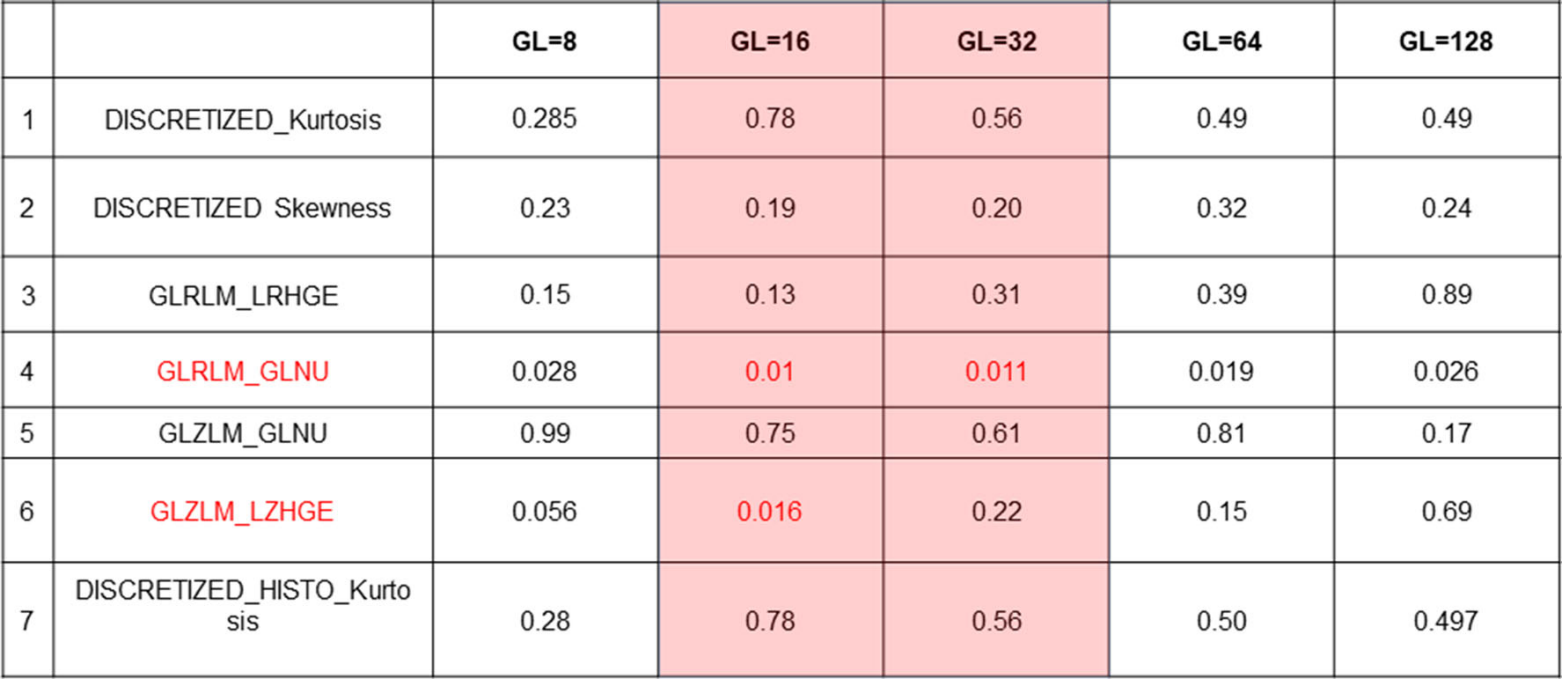
Univariate logistic regression analysis. *P*-values are given for the remaining 7 radiomic features and for each of the 5 Gy levels (GL). Significant univariate predictors of decreased myocardial flow reserve after application of the Benjamini–Hochberg procedure to correct for multiple comparisons at a 10% false discovery rate are depicted in red. Only two GL (16 and 32) exhibited significant features

A univariate regression analysis demonstrated that among all 5 GL, only GL = 16 and GL = 32 included significant predictors of decreased MFR (Figure 5). Hence, all other GLs were excluded (GL = 8, GL = 64 and GL = 128). The univariate significant predictors of GL = 16 were GLRLM_GLNU and GLZLM_LZHGE (all *P* ≤ .016), while in GL = 32 only GLRLM_GLNU was a significant predictor (*P* = .011). Therefore, the final analysis was performed on GL = 16 (Figure 5).

### Predictive power

**Figure 6:**
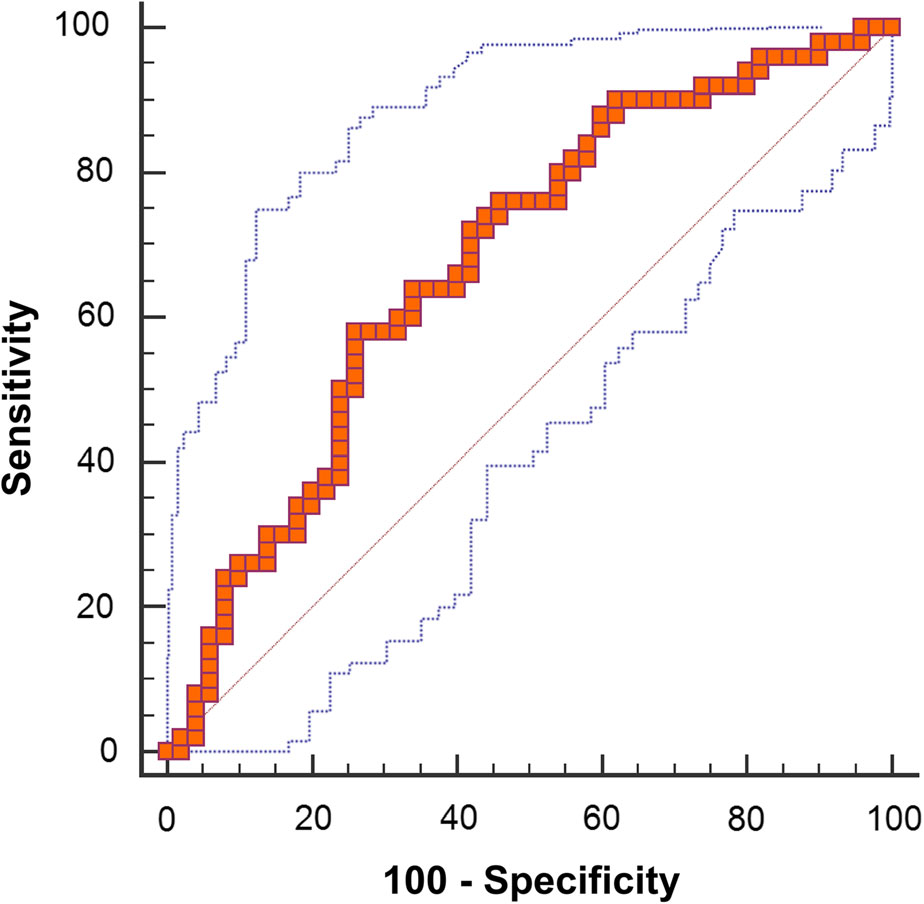
Receiver operating curve for GLRLM_GLNU derived from 16 GL discretization for the prediction of decreased MFR in patients with visually normal MPI

In multivariate regression analysis with GLRLM_GLNU and GLZLM_LZHGE, only GLRLM_GLNU remained in the model and at the optimal cut-off of ≤ 948 could differentiate patients with preserved versus decreased MFR with an accuracy of 67%, a sensitivity of 74%, specificity of 58%, a negative predictive value of 64%, and a positive predictive value of 69% (Figure 6).

Application of GLRLM_GLNU to the validation cohort, using a cut-off of 948 yielded a sensitivity of 87%, specificity of 27%, negative predictive value of 54% and a positive predictive value of 67%.

The results from an alternative feature selection process performed for each GL separately are provided in the supplemental material. Of note, even if a different approach to the feature selection process was applied and performed separately for each GL, GLRLM_GLNU remained the only significant independent predictor for a decreased MFR.

## Discussion

In this proof-of-concept study, using 13N-ammonia PET MPI, we demonstrate the value of radiomic features extracted from normal retention images to identify patients with globally reduced MFR.

The principle of radiomics analysis builds on the extraction of voxel values, their relationship to each other as well as texture patterns from the image, providing quantitative characteristics of medical images which are imperceptible to the human eye. Radiomics have been extensively studied in oncoradiology, where they exhibit the ability to capture tumor heterogeneity even at cellular level and are strong prognostic determinants of patients’ survival.^21,22^ In the cardiovascular domain, the application of radiomics is a relatively new direction and its full potential is yet to be explored. While several studies have already demonstrated the value of radiomics and machine learning applications in the imaging of atherosclerosis, perivascular adipose tissue phenotyping, as well as detection of cardiac pathologies with different cardiac imaging modalities such as cardiac CT and magnetic resonance (CMR) imaging,^14,23–25^ comparable studies for nuclear cardiology, and in particular for nuclear MPI are scarce. To the best of our knowledge, only one study by Edalat-Javid et al. has yet applied the concept of radiomics to nuclear MPI aiming to assess repeatability of various radiomic features.^26^

In our study, a single radiomic feature (GLRLM_GLNU) extracted from visually normal retention PET MPI was independently predicting reduced MFR albeit with moderate accuracy. GLRLM_GLNU is a measure of non-uniformity within the GL run length matrix (GLRLM) and therefore represents heterogeneity of the image. Interestingly, this same parameter has already shown value, robustness and reproducibility in CMR studies.^27–29^ GLRLM_GLNU characterises variability of GL intensity values in the image, for which lower values indicate homogeneity, while higher values characterize more heterogeneous intensity patterns. It is important to understand the characteristics and nature of radiomic features because within a statistical setting prone to false discoveries due to multiple comparisons, plausibility of a finding becomes crucially important. It seems logical and likely that a feature associated with subtle heterogeneities of tracer retention exhibits predictive power to identify underlying and diffuse MBF restrictions. This plausibility emphasizes the potential of the radiomics approach for other applications of nuclear cardiology.

Naturally, the results from this study do not currently confer direct clinical implications given the fact that PET MPI is a quantitative imaging modality which per se allows for the detection of diffuse MBF impairment. Our results, however, validate the basic concept of such an approach for the nuclear cardiology domain and the simplistic algorithm presented here, namely, the use of readily available polar plots from clinical routine as a basis for radiomics analysis should be recognized as a strength of the concept presented here and may pave the way for initiation of future studies aiming to identify radiomics extracted from conventional SPECT MPI to predict diffusely impaired blood flow—preferably with a robust standard of reference. It has to be noted that, on its own, the diagnostic accuracy of the radiomic feature presented here is only moderate and future studies should also focus on the value of radiomics as an adjunct to other clinical and imaging parameters such as the coronary artery calcium score.

### Limitations and strengths

Several limitations should be acknowledged. First, given the nature of texture analysis algorithms our results may not be applicable to PET MPI acquired on scanners of other vendors, reconstructed with other algorithms or performed with different tracers. Interestingly, however, different types of PET scanners were used for the acquisition of PET MPI in the present study, corroborating the robustness of our findings. On the other hand, due to the very asymmetric distribution of the population among the various scanners, we were unable to perform a subanalysis providing more insight into the potential scanner dependent differences in the radiomics’ performance. Furthermore, we included only radiomics features made available by the LifeX software which may not cover the entire spectrum of possible parameters. It may be possible that other features not addressed by this work may have diagnostic value as well. On the other hand, the use of LifeX for radiomic feature extraction constitutes also a strength of the present study because its speed and ease of use, particularly in light of our simplified methodology, relying only on polar plots as generated in every-day clinical routine.

## Conclusion

A single radiomic feature (GLRLM_GLNU) extracted from visually normal 13N-ammonia PET retention images independently predicts reduced global MFR with moderate accuracy. This concept could potentially be applied to other myocardial perfusion imaging modalities based purely on relative distribution patterns to allow for better detection of diffuse disease.

## New knowledge gained

Radiomics analysis from 13N-ammonia PET retention images is feasible and a single radiomic predicts globally reduced MFR in patients with visually normal 13N-ammonia PET retention images.

## Supplementary Information

Below is the link to the electronic supplementary material.

Supplementary file1 (DOCX 4228 kb)

Supplementary file2 (MP3 3199 kb)

Supplementary file3 (PPTX 1738 kb)

## Abbreviations

AUC: Area under the curve
CAD: Coronary artery disease
GL: Gray-level
FDR: False discovery rate
MBF: Myocardial blood flow
MFR: Myocardial flow reserve
MPI: Myocardial perfusion imaging
PET: Positron emission tomography
ROC: Receiver operating curve
SPECT: Single-photon emission computed tomography
