# Supplementary material for: Radiomics for the detection of diffusely impaired myocardial perfusion: A proof-of-concept study using 13N-ammonia positron emission tomography
Source: J Nucl Cardiol. 2023 Jan 5;30(4):1474–83. doi: 10.1007/s12350-022-03179-y (PMC10371953; doi:10.1007/s12350-022-03179-y)
Supplement: Supplementary file 1 — Supplementary file1 (DOCX 4228 kb) [file 12350_2022_3179_MOESM1_ESM.docx]

**Supplementary material**





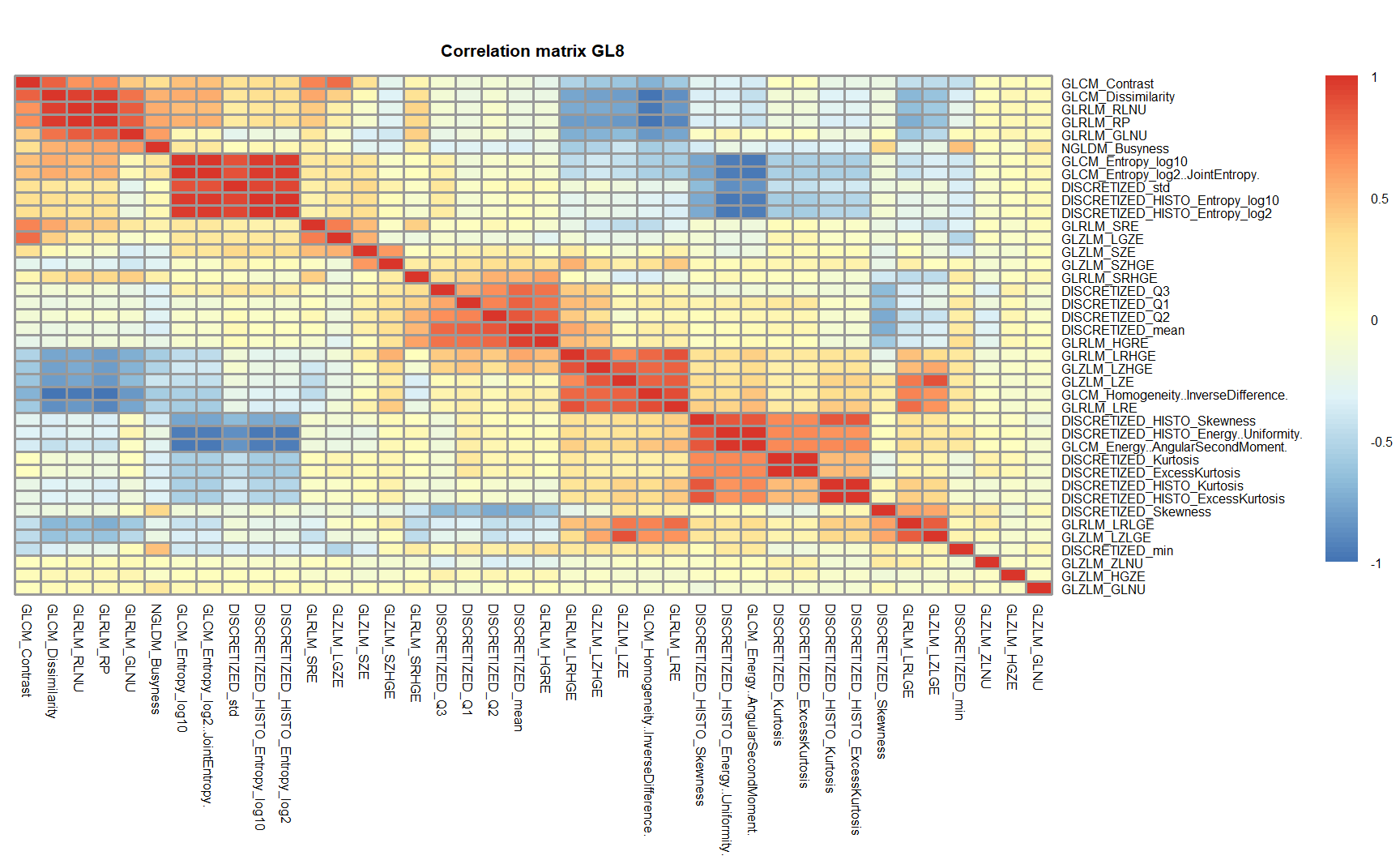


**Figure S1.** Heatmap of pairwise Pearson’s correlation between radiomic features for GL 8.


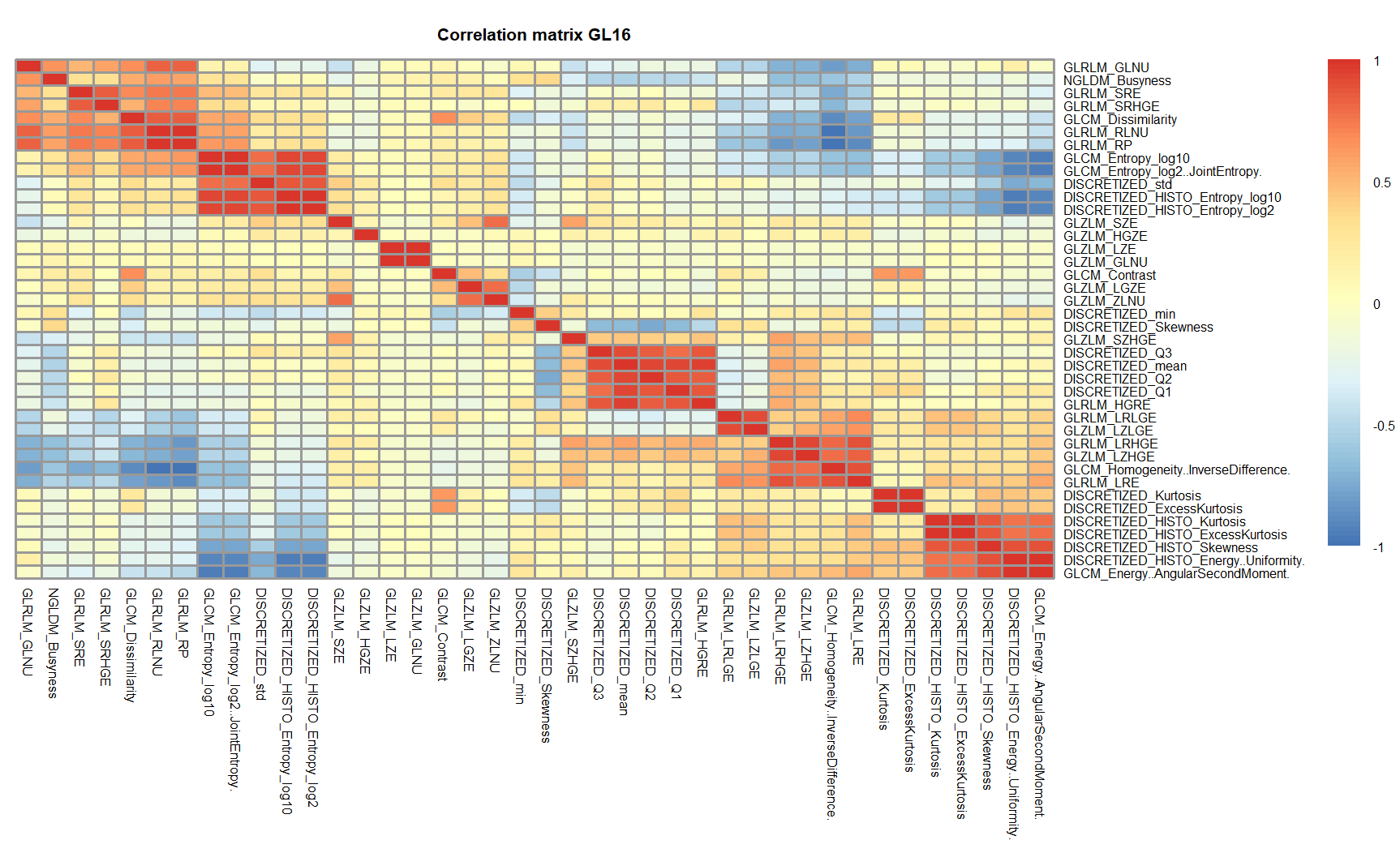


**Figure S2.** Heatmap of pairwise Pearson’s correlation between radiomic features for GL 16.


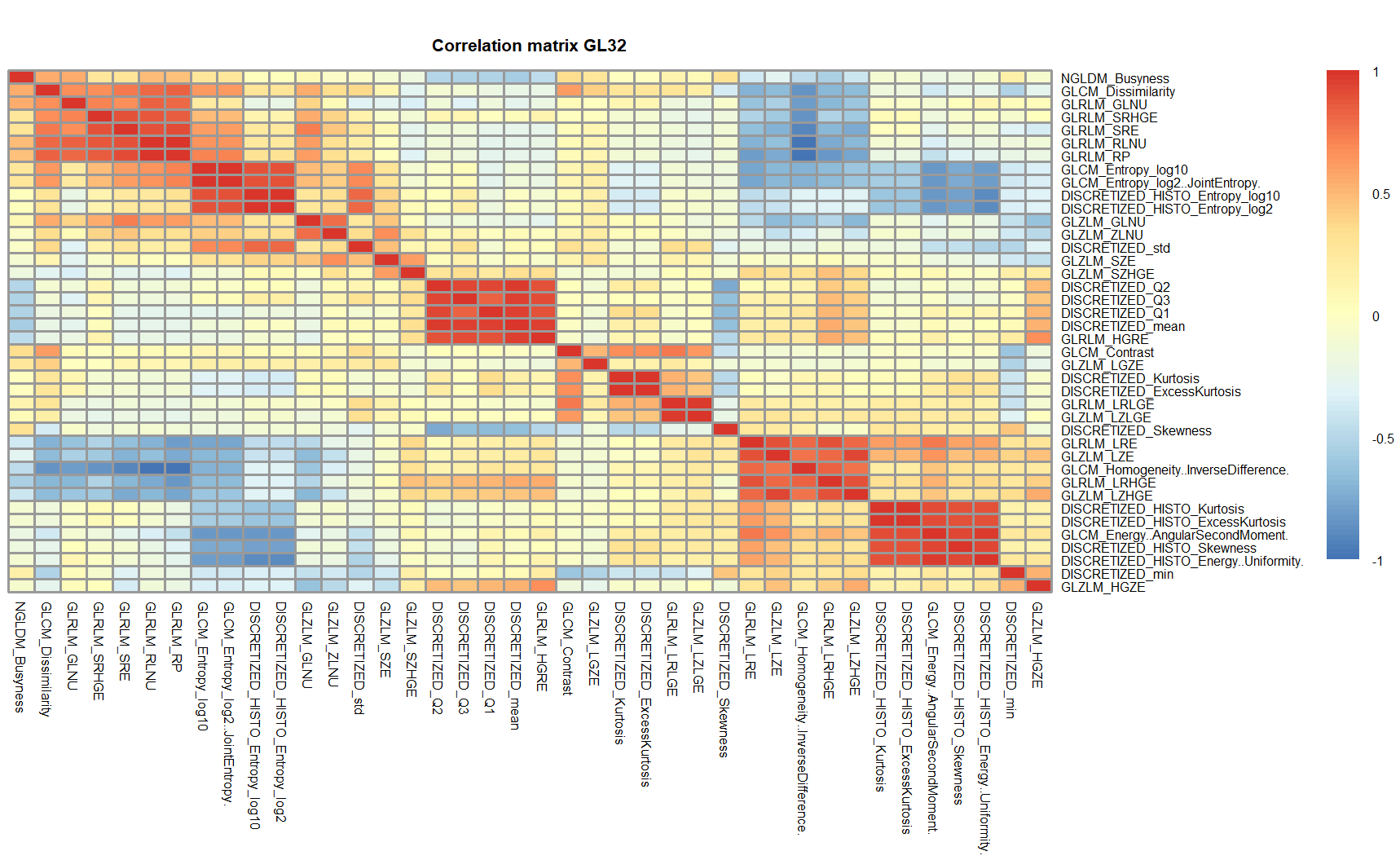


**Figure S3.** Heatmap of pairwise Pearson’s correlation between radiomic features for GL 32.

**Figure S4.** Heatmap of pairwise Pearson’s correlation between radiomic features for GL 64.


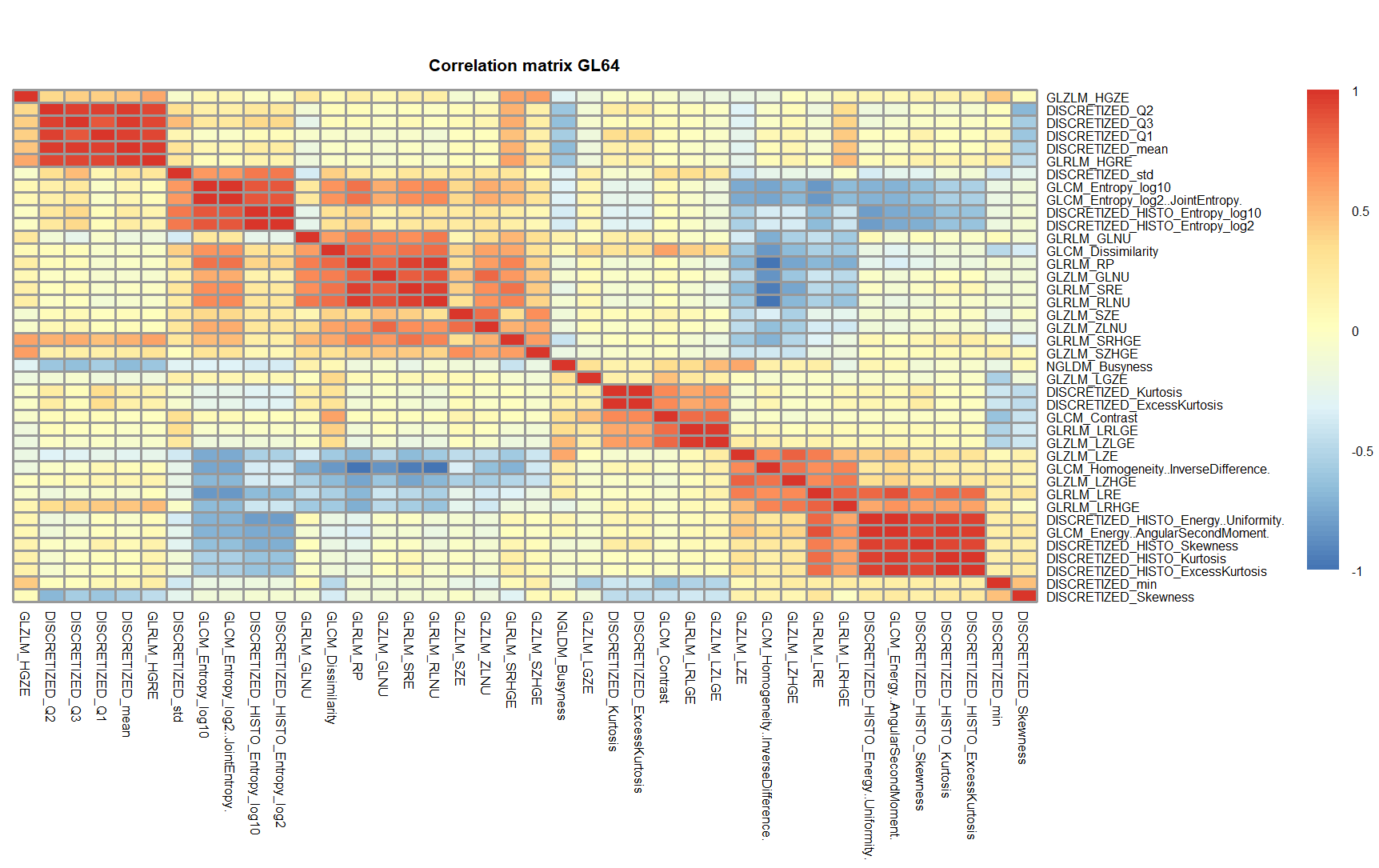

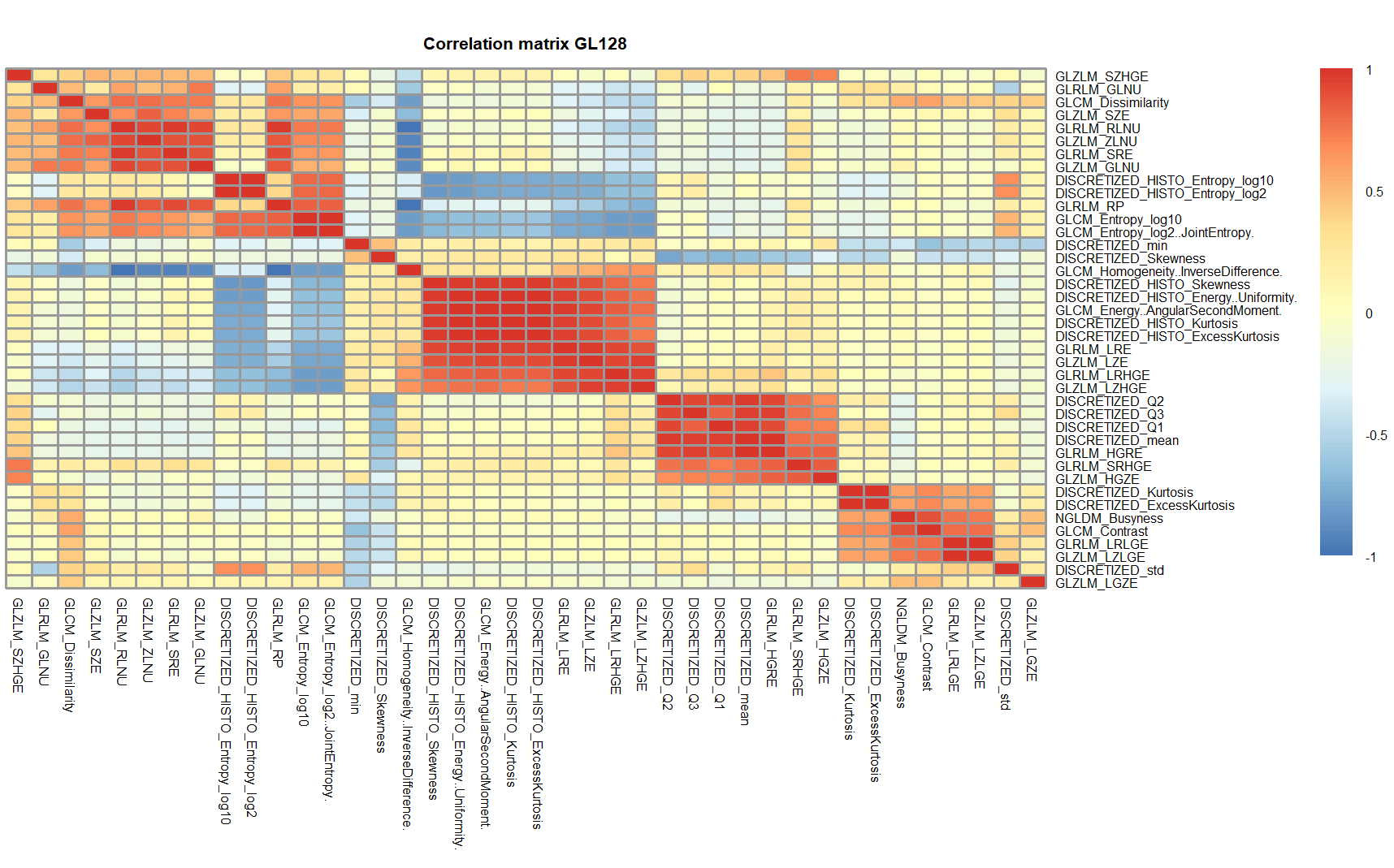


**Figure S5.** Heatmap of pairwise Pearson’s correlation between radiomic features for GL 128.


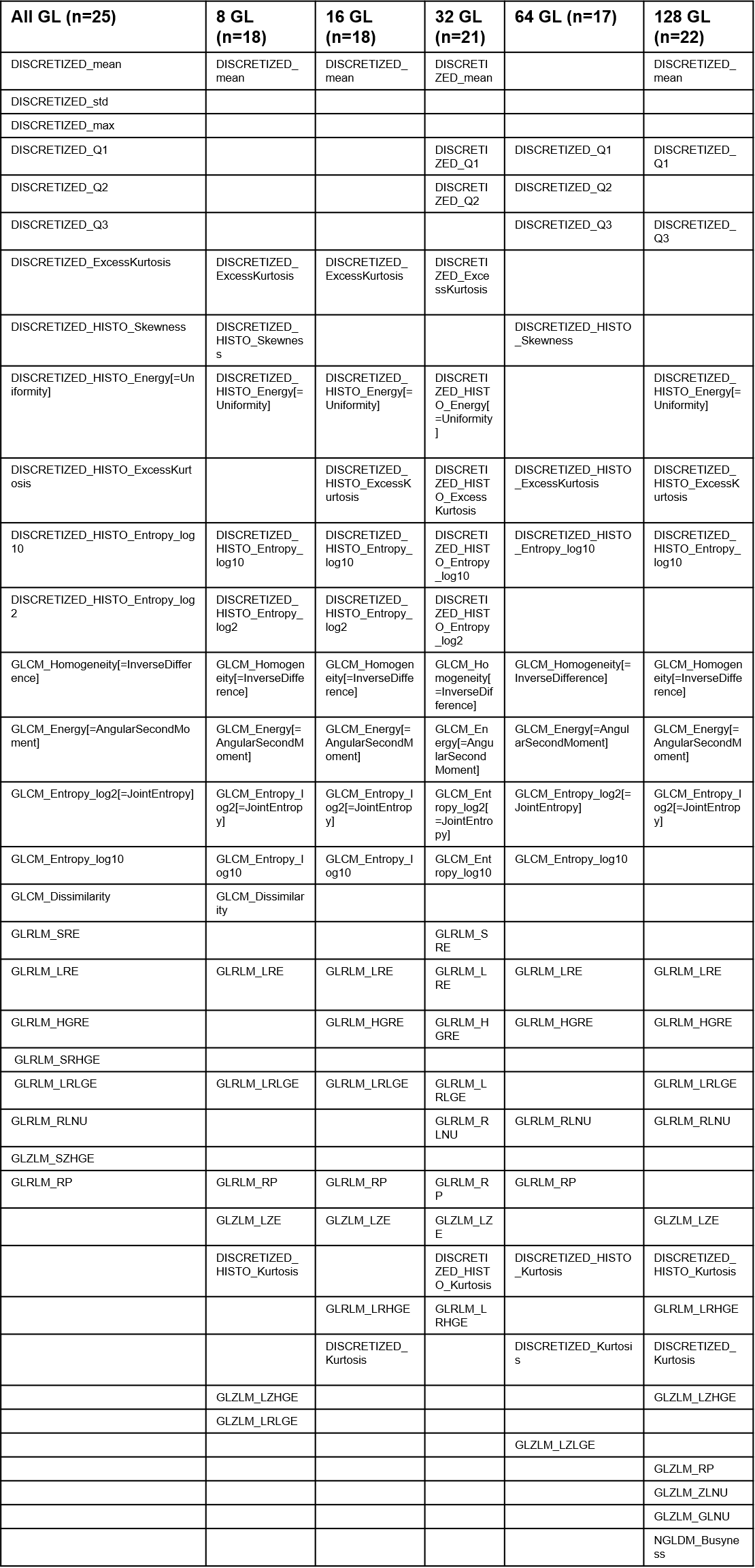


**Figure S6.** Features with a pairwise Pearson’s correlation coefficient |𝜌| ≥ 0.85 for each GL discretization.


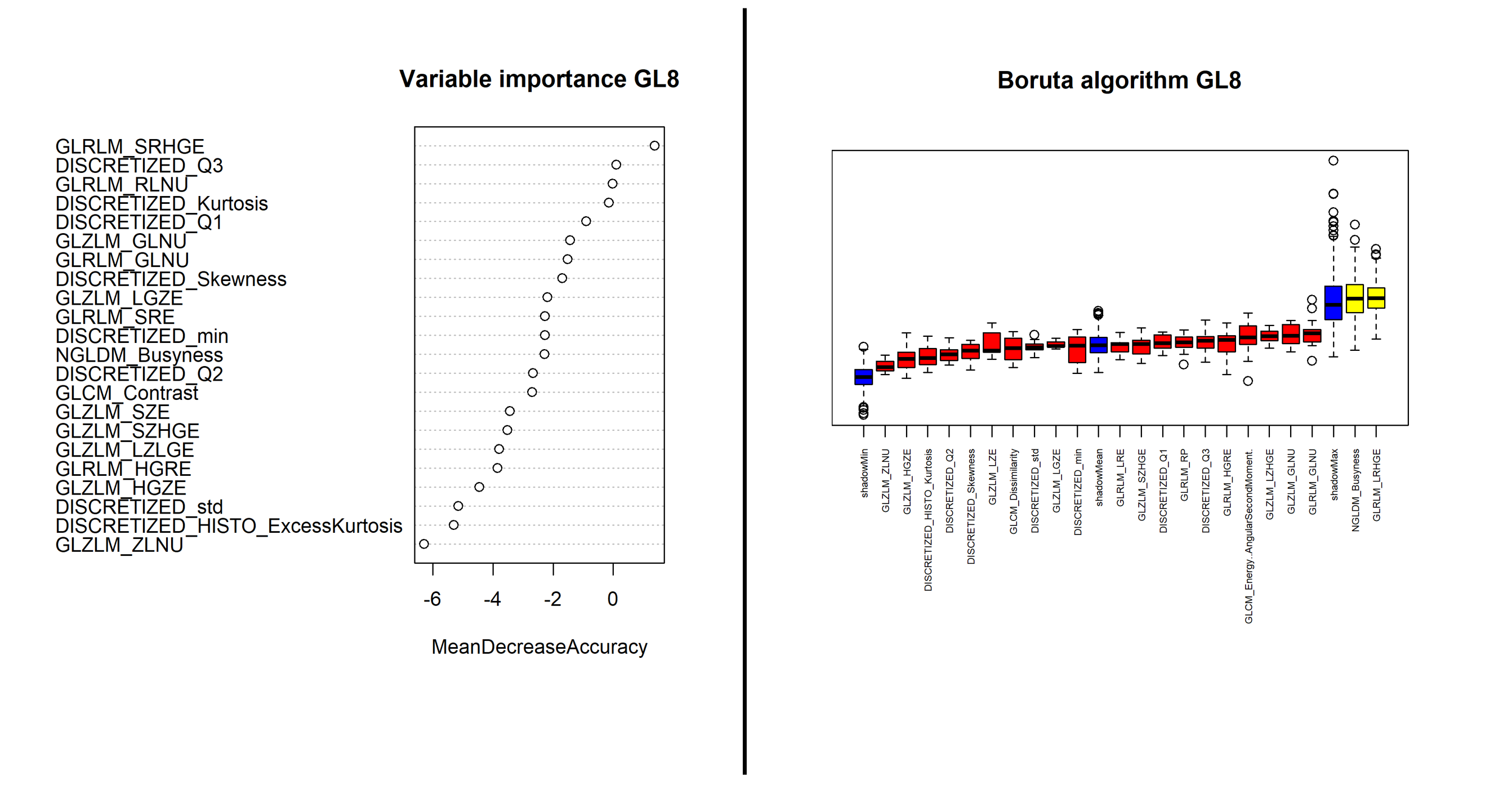


**Figure S7.** Feature selection with variable importance plot and Boruta algorithm for GL8. Blue boxes correspond to shadow attributes, green color indicates important attributes, and red boxes are unimportant.


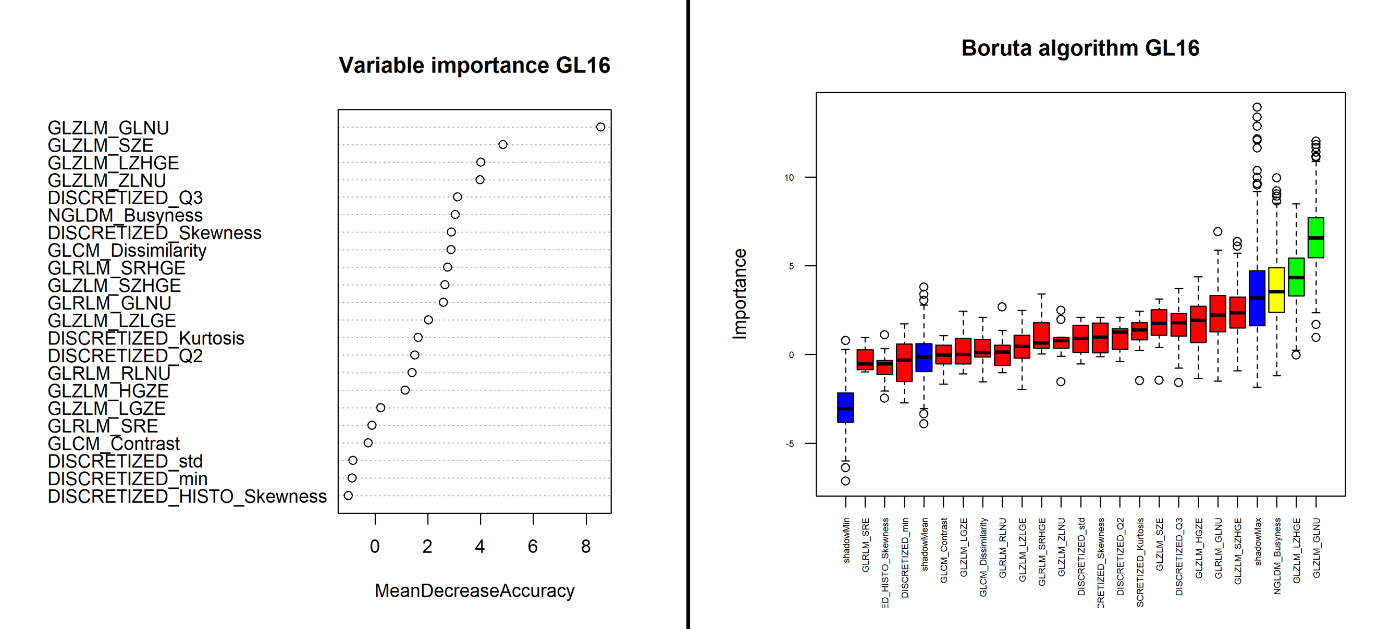


**Figure S8.** Feature selection with variable importance plot and Boruta algorithm for GL16.


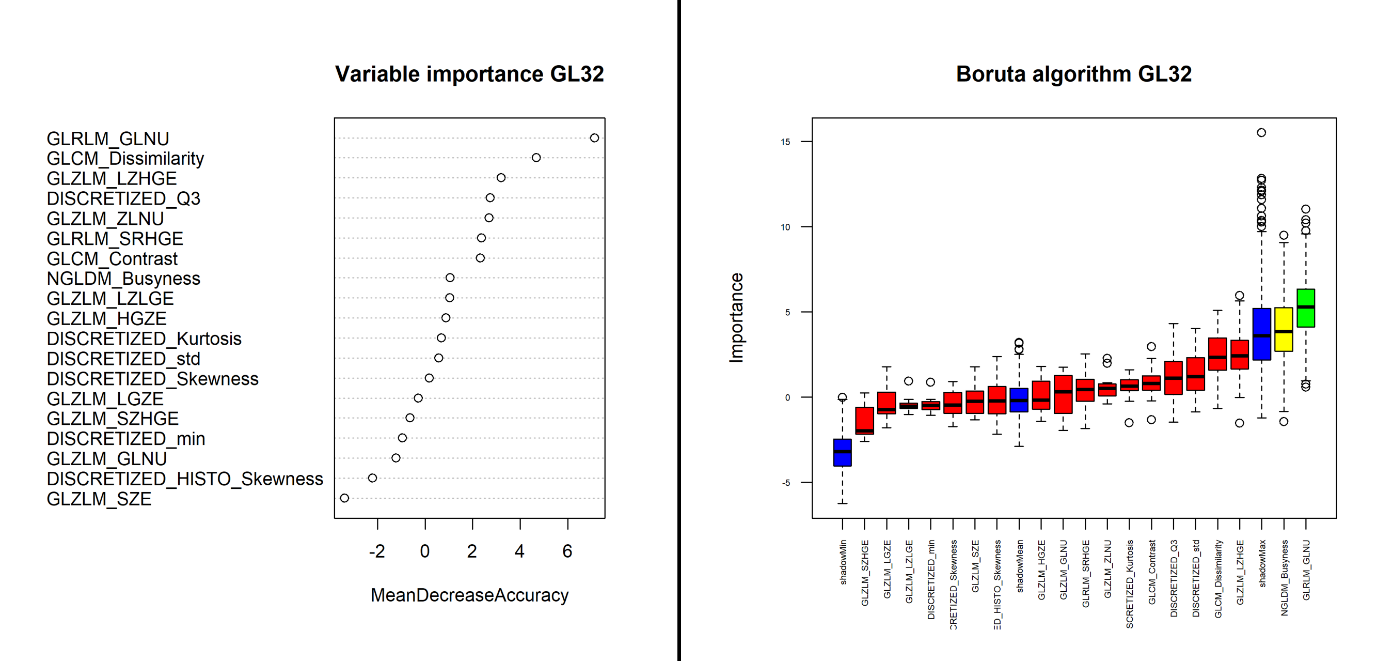


**Figure S9.** Feature selection with variable importance plot and Boruta algorithm for GL32.


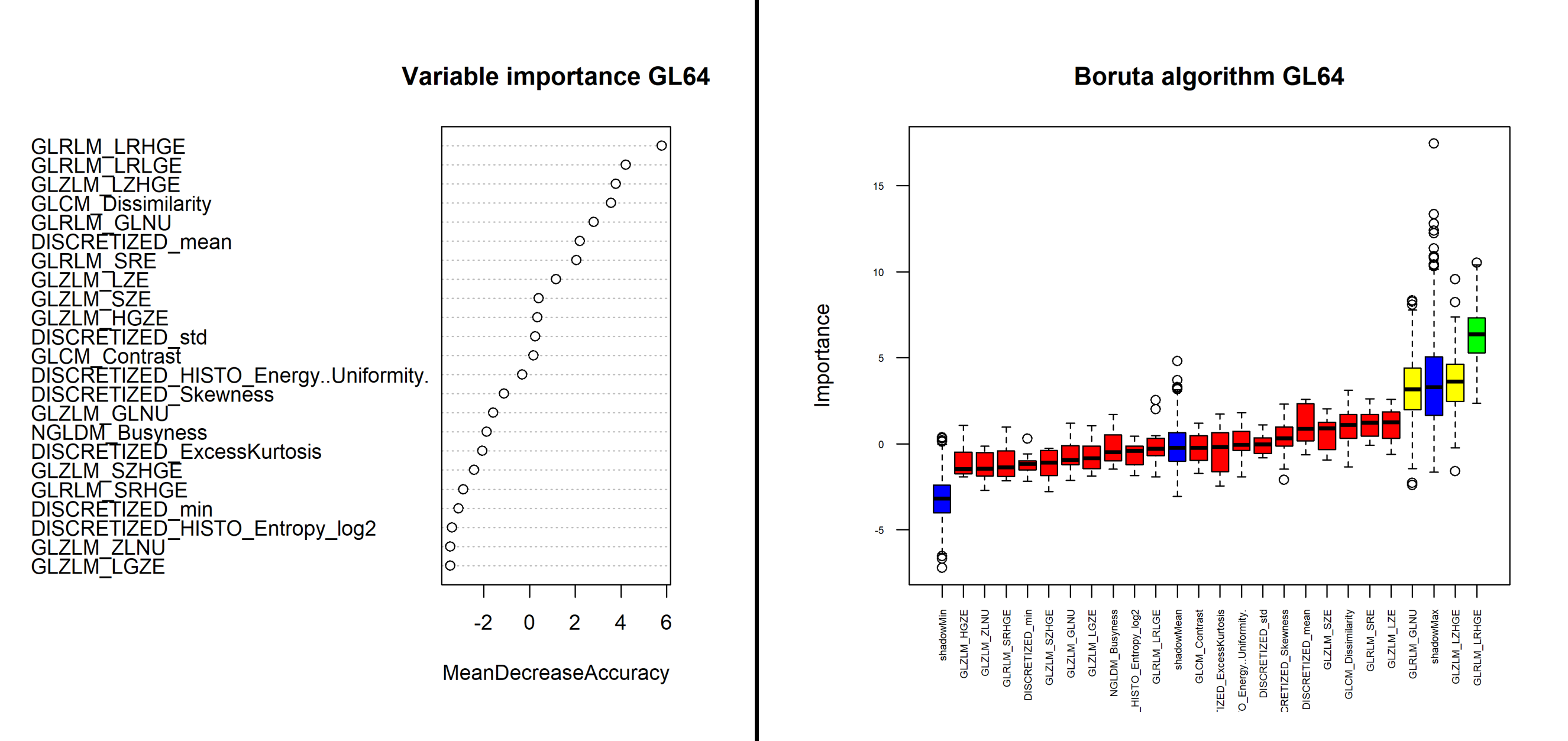


**Figure S10.** Feature selection with variable importance plot and Boruta algorithm for GL64.


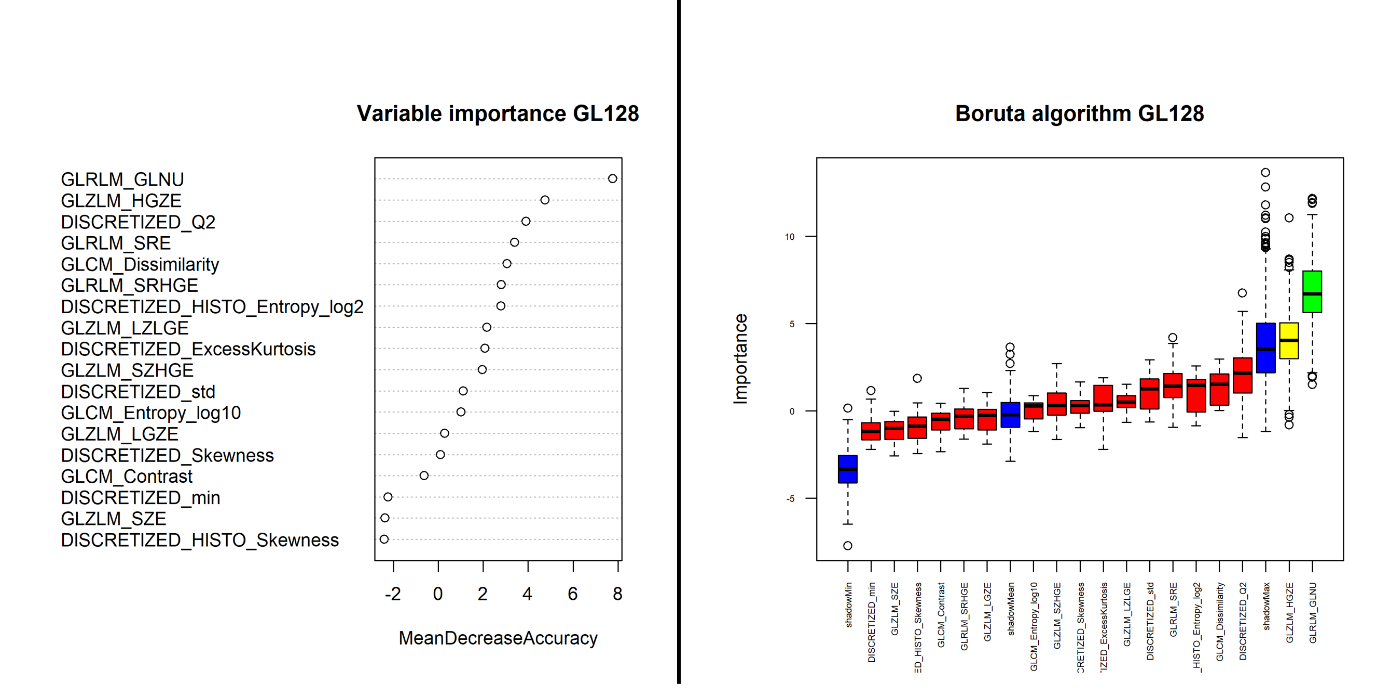


**Figure S11.** Feature selection with variable importance plot and Boruta algorithm for GL128.





**Figure S12.** Results from the univariate and multivariate regression analysis including all the variables from variable importance plot GL16 (see Figure S8 above) with numerical estimate of the feature importance >0. Of note, and as in the approach in the main manuscript, only GLRM_GLNU remained a significant predictor of reduced MFR in multivariate analysis.
