# Supplementary material for: Radiomics for the detection of diffusely impaired myocardial perfusion: A proof-of-concept study using 13N-ammonia positron emission tomography
Source: J Nucl Cardiol. 2023 Jan 5;30(4):1474–83. doi: 10.1007/s12350-022-03179-y (PMC10371953; doi:10.1007/s12350-022-03179-y)
Supplement: Supplementary file 3 — Supplementary file3 (PPTX 1738 kb) [file 12350_2022_3179_MOESM3_ESM.pptx]

## Slide 1
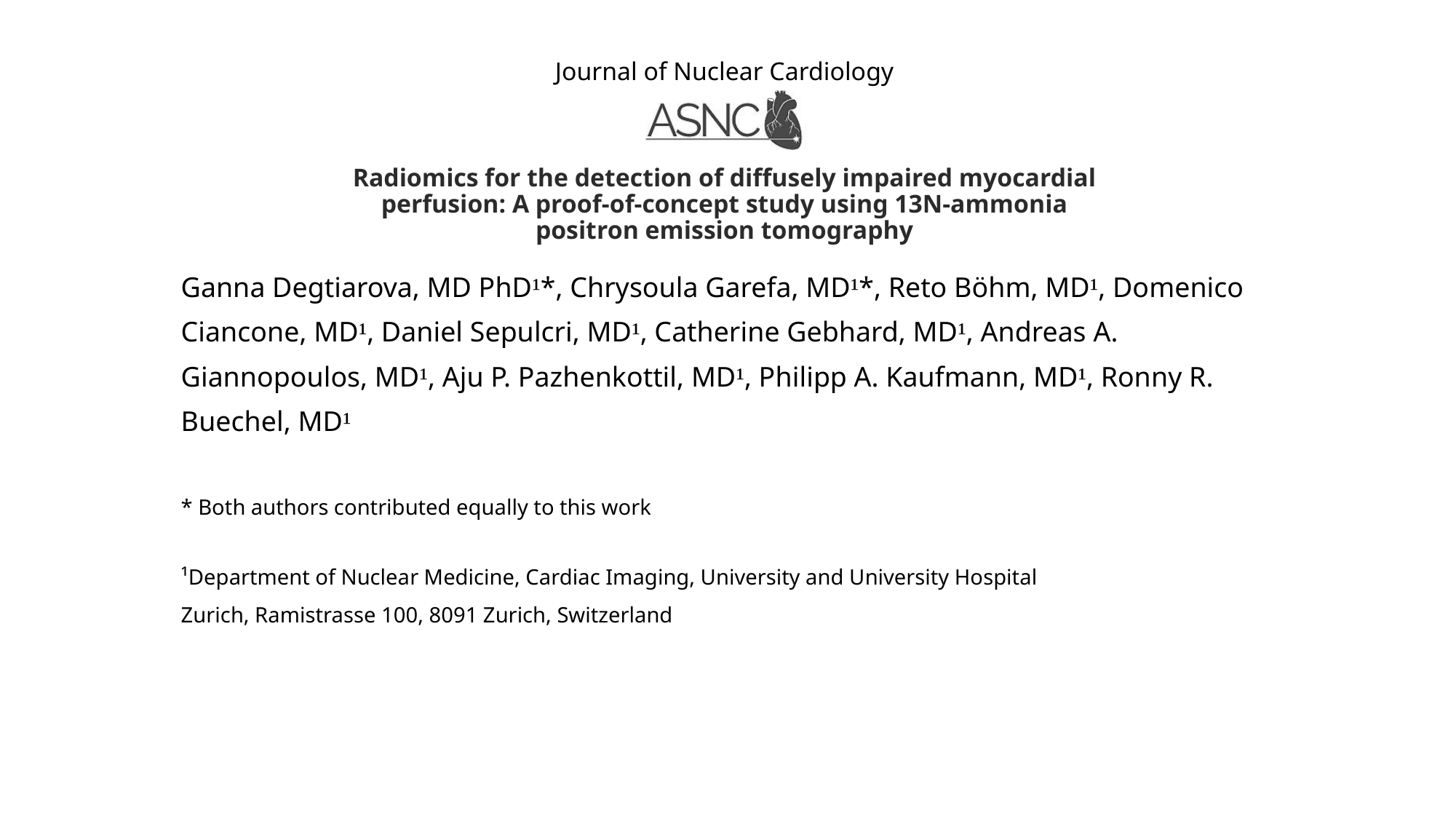

# Journal of Nuclear Cardiology Radiomics for the detection of diffusely impaired myocardial perfusion: A proof-of-concept study using 13N-ammonia positron emission tomography
Ganna Degtiarova, MD PhD¹*, Chrysoula Garefa, MD¹*, Reto Böhm, MD¹, Domenico
Ciancone, MD¹, Daniel Sepulcri, MD¹, Catherine Gebhard, MD¹, Andreas A.
Giannopoulos, MD¹, Aju P. Pazhenkottil, MD¹, Philipp A. Kaufmann, MD¹, Ronny R.
Buechel, MD¹
* Both authors contributed equally to this work
¹Department of Nuclear Medicine, Cardiac Imaging, University and University Hospital
Zurich, Ramistrasse 100, 8091 Zurich, Switzerland

## Slide 2
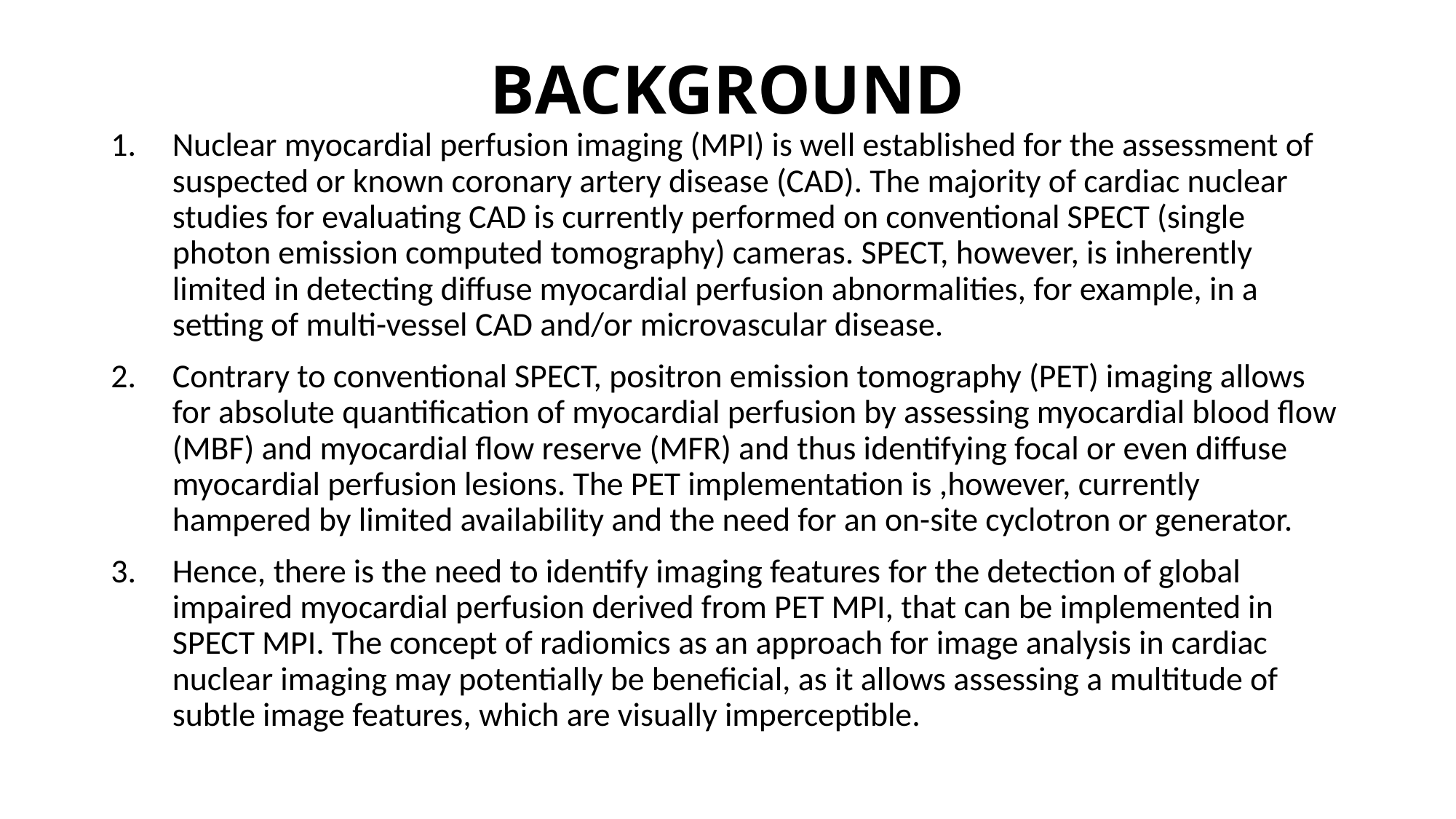

# BACKGROUND
Nuclear myocardial perfusion imaging (MPI) is well established for the assessment of suspected or known coronary artery disease (CAD). The majority of cardiac nuclear studies for evaluating CAD is currently performed on conventional SPECT (single photon emission computed tomography) cameras. SPECT, however, is inherently limited in detecting diffuse myocardial perfusion abnormalities, for example, in a setting of multi-vessel CAD and/or microvascular disease.
Contrary to conventional SPECT, positron emission tomography (PET) imaging allows for absolute quantification of myocardial perfusion by assessing myocardial blood flow (MBF) and myocardial flow reserve (MFR) and thus identifying focal or even diffuse myocardial perfusion lesions. The PET implementation is ,however, currently hampered by limited availability and the need for an on-site cyclotron or generator.
Hence, there is the need to identify imaging features for the detection of global impaired myocardial perfusion derived from PET MPI, that can be implemented in SPECT MPI. The concept of radiomics as an approach for image analysis in cardiac nuclear imaging may potentially be beneficial, as it allows assessing a multitude of subtle image features, which are visually imperceptible.

## Slide 3
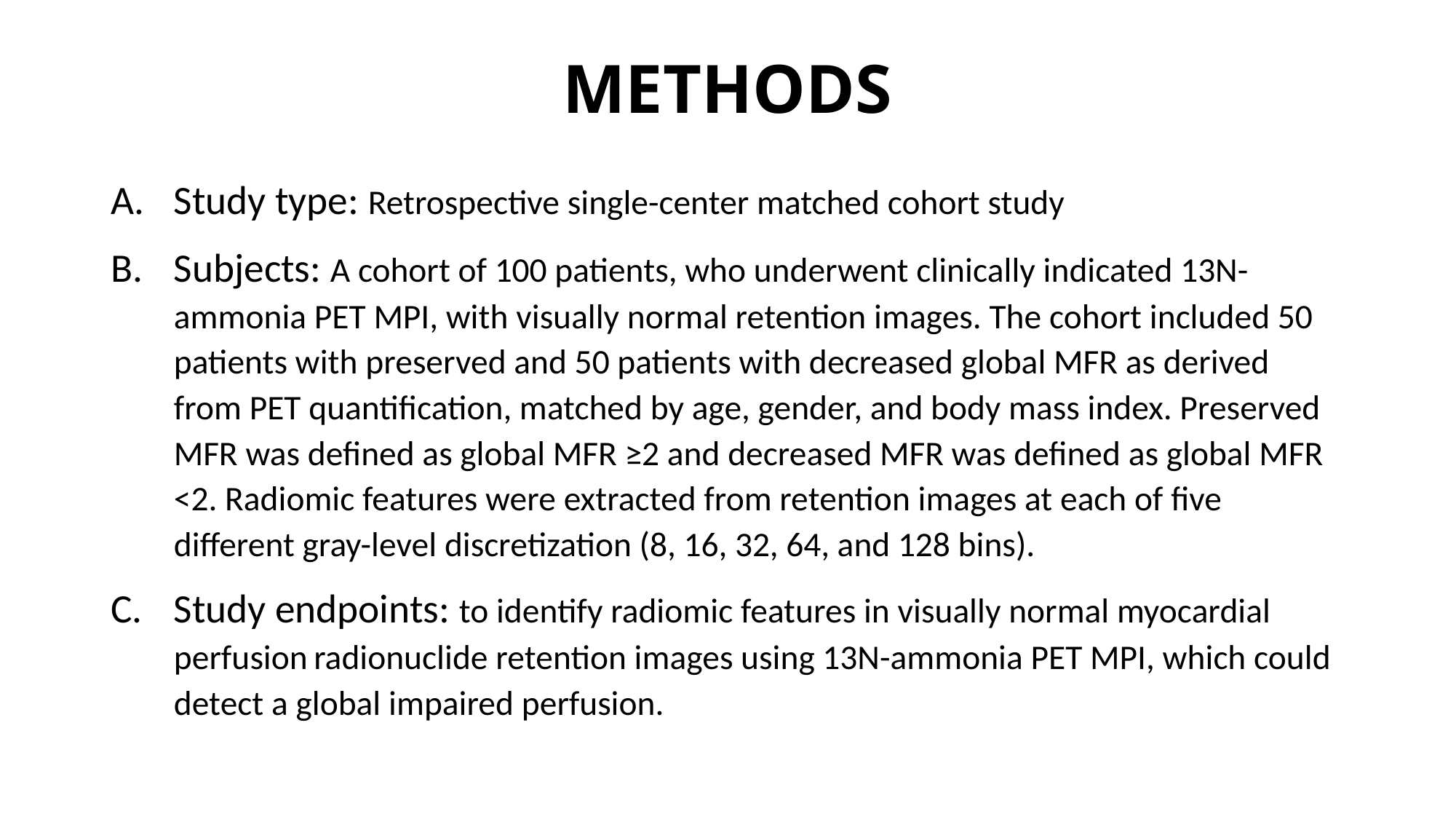

# METHODS
Study type: Retrospective single-center matched cohort study
Subjects: A cohort of 100 patients, who underwent clinically indicated 13N-ammonia PET MPI, with visually normal retention images. The cohort included 50 patients with preserved and 50 patients with decreased global MFR as derived from PET quantification, matched by age, gender, and body mass index. Preserved MFR was defined as global MFR ≥2 and decreased MFR was defined as global MFR <2. Radiomic features were extracted from retention images at each of five different gray-level discretization (8, 16, 32, 64, and 128 bins).
Study endpoints: to identify radiomic features in visually normal myocardial perfusion radionuclide retention images using 13N-ammonia PET MPI, which could detect a global impaired perfusion.

## Slide 4
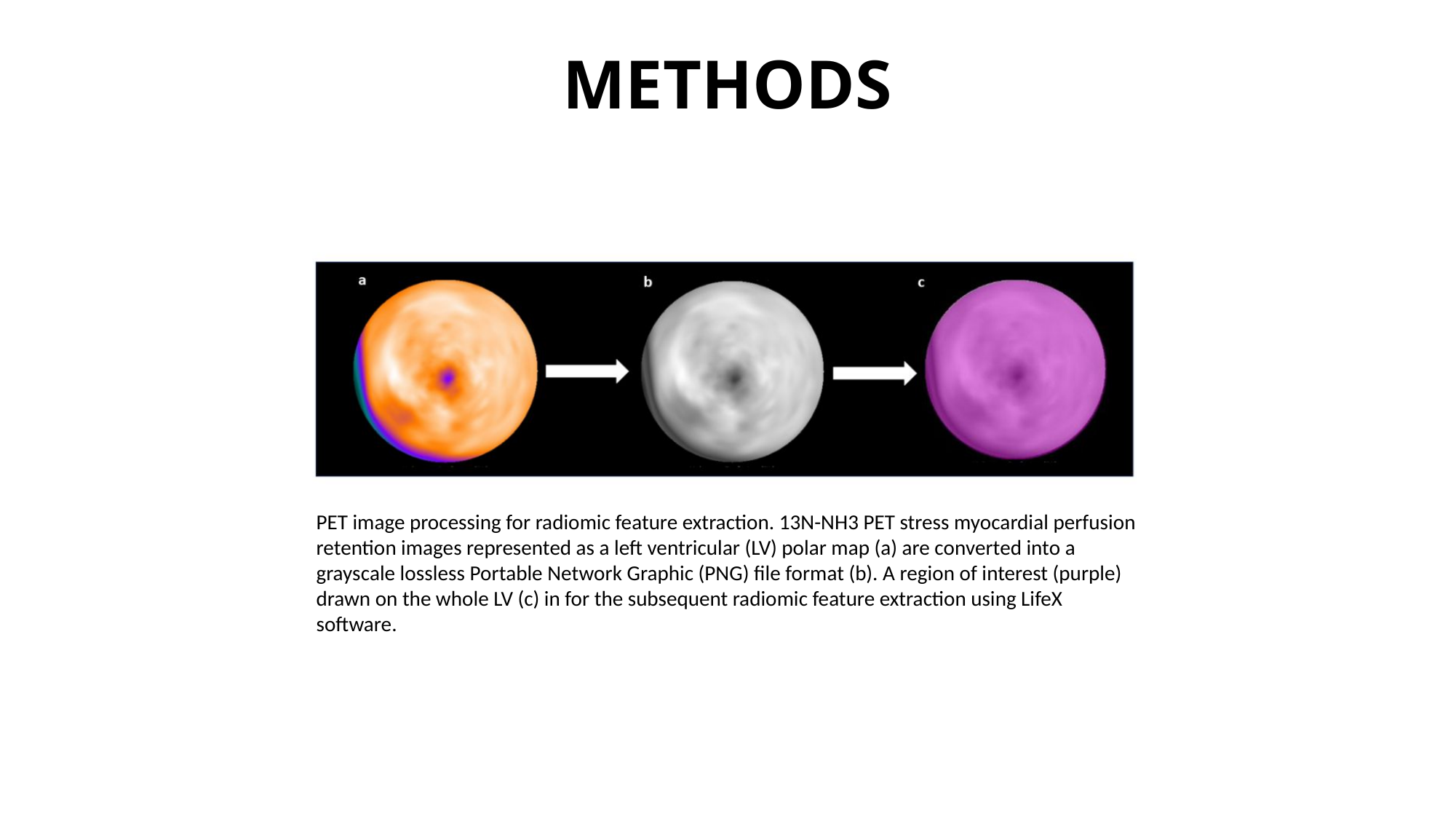

# METHODS
PET image processing for radiomic feature extraction. 13N-NH3 PET stress myocardial perfusion
retention images represented as a left ventricular (LV) polar map (a) are converted into a grayscale lossless Portable Network Graphic (PNG) file format (b). A region of interest (purple) drawn on the whole LV (c) in for the subsequent radiomic feature extraction using LifeX software.

## Slide 5
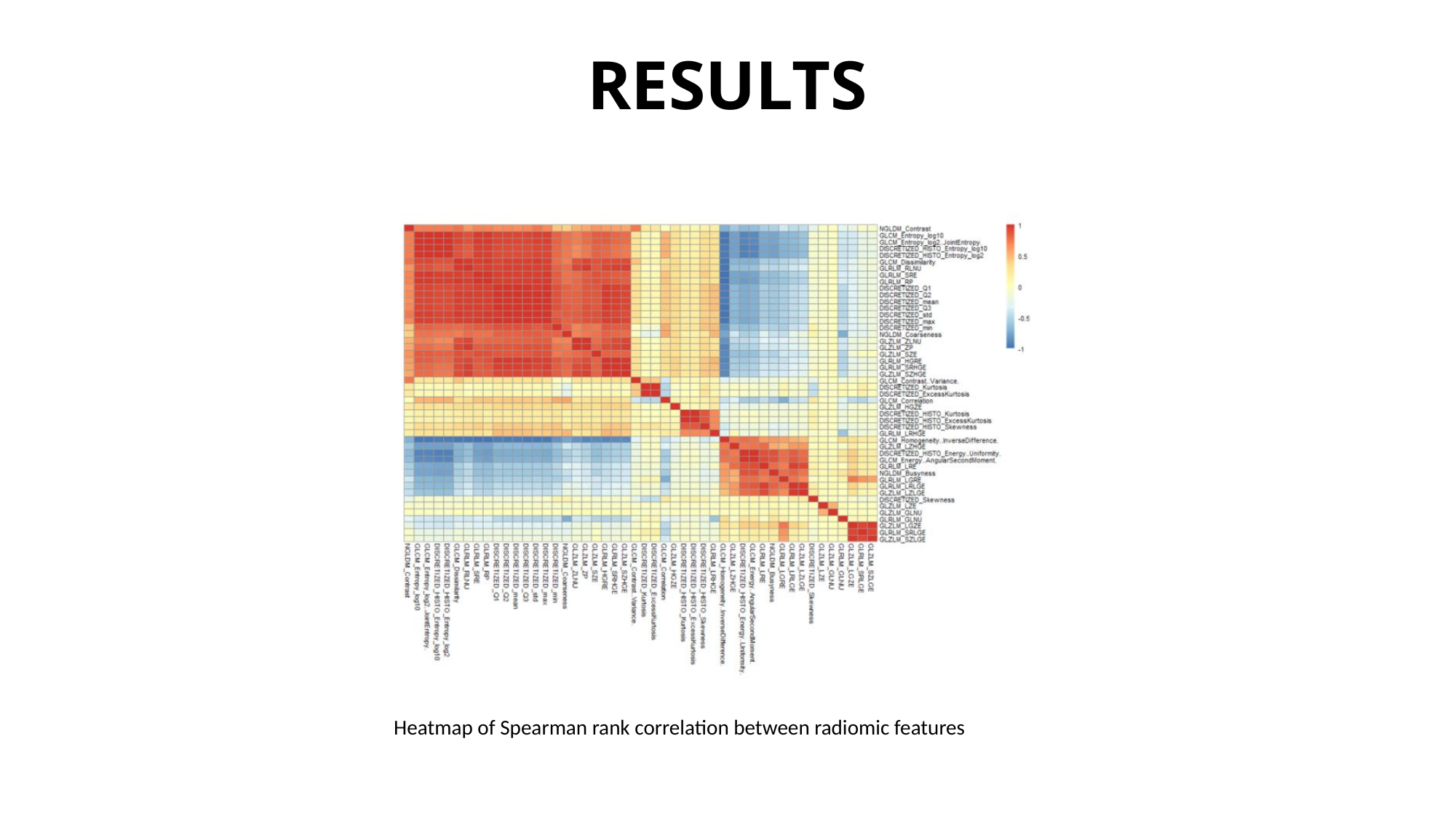

# RESULTS
Heatmap of Spearman rank correlation between radiomic features

## Slide 6
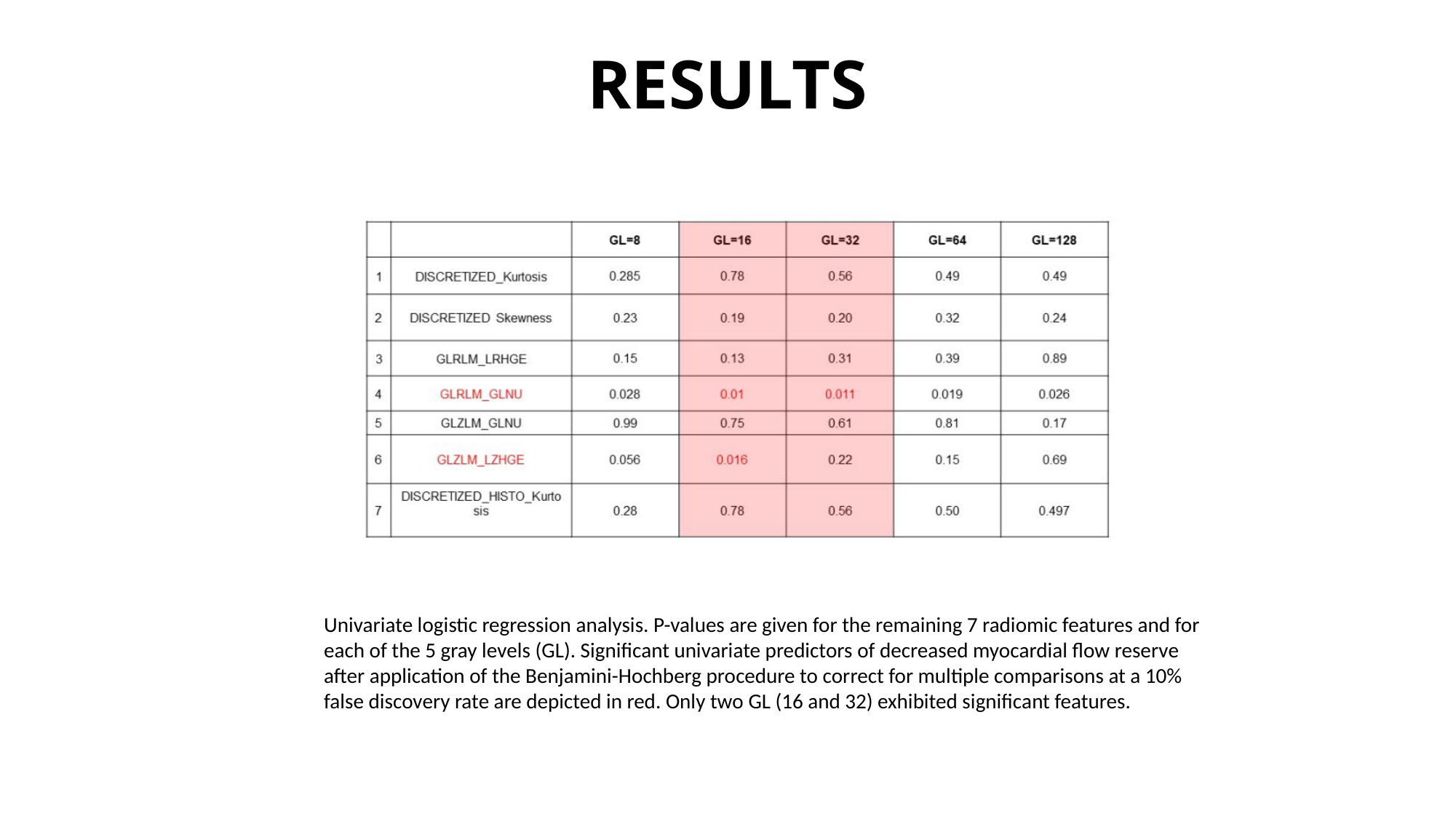

# RESULTS
Univariate logistic regression analysis. P-values are given for the remaining 7 radiomic features and for each of the 5 gray levels (GL). Significant univariate predictors of decreased myocardial flow reserve after application of the Benjamini-Hochberg procedure to correct for multiple comparisons at a 10% false discovery rate are depicted in red. Only two GL (16 and 32) exhibited significant features.

## Slide 7
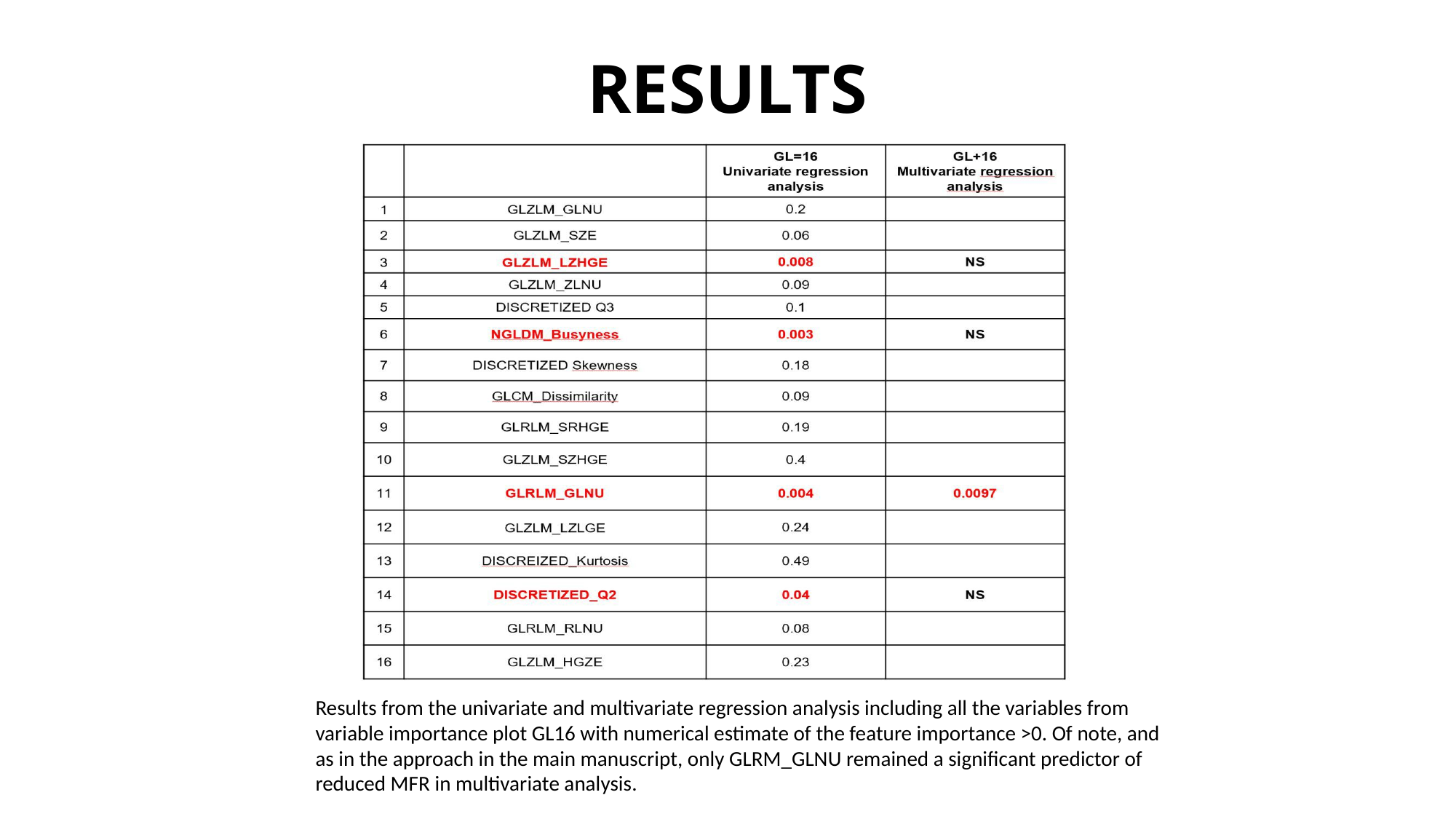

# RESULTS
Results from the univariate and multivariate regression analysis including all the variables from variable importance plot GL16 with numerical estimate of the feature importance >0. Of note, and as in the approach in the main manuscript, only GLRM_GLNU remained a significant predictor of reduced MFR in multivariate analysis.

## Slide 8
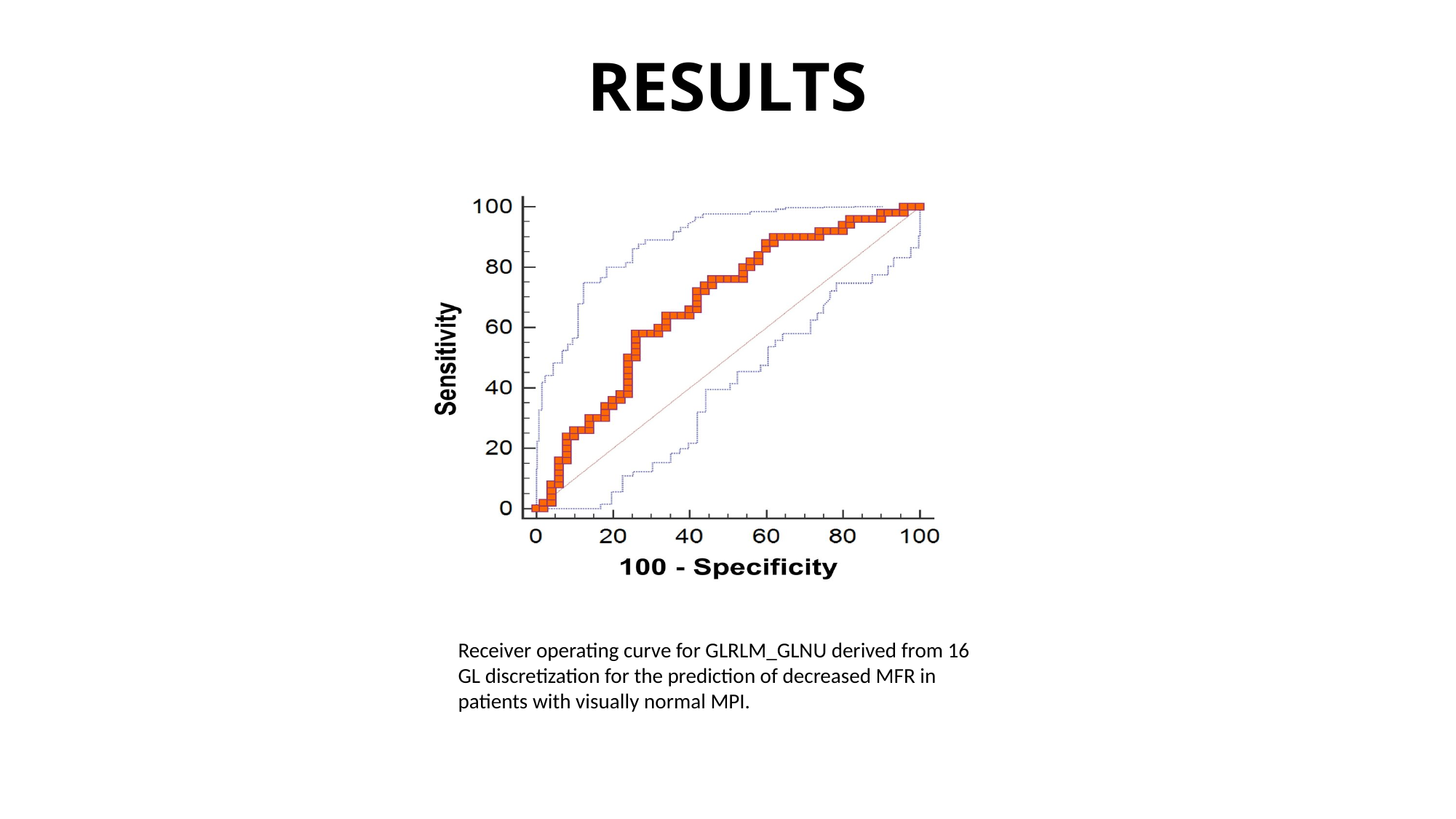

# RESULTS
Receiver operating curve for GLRLM_GLNU derived from 16 GL discretization for the prediction of decreased MFR in patients with visually normal MPI.

## Slide 9
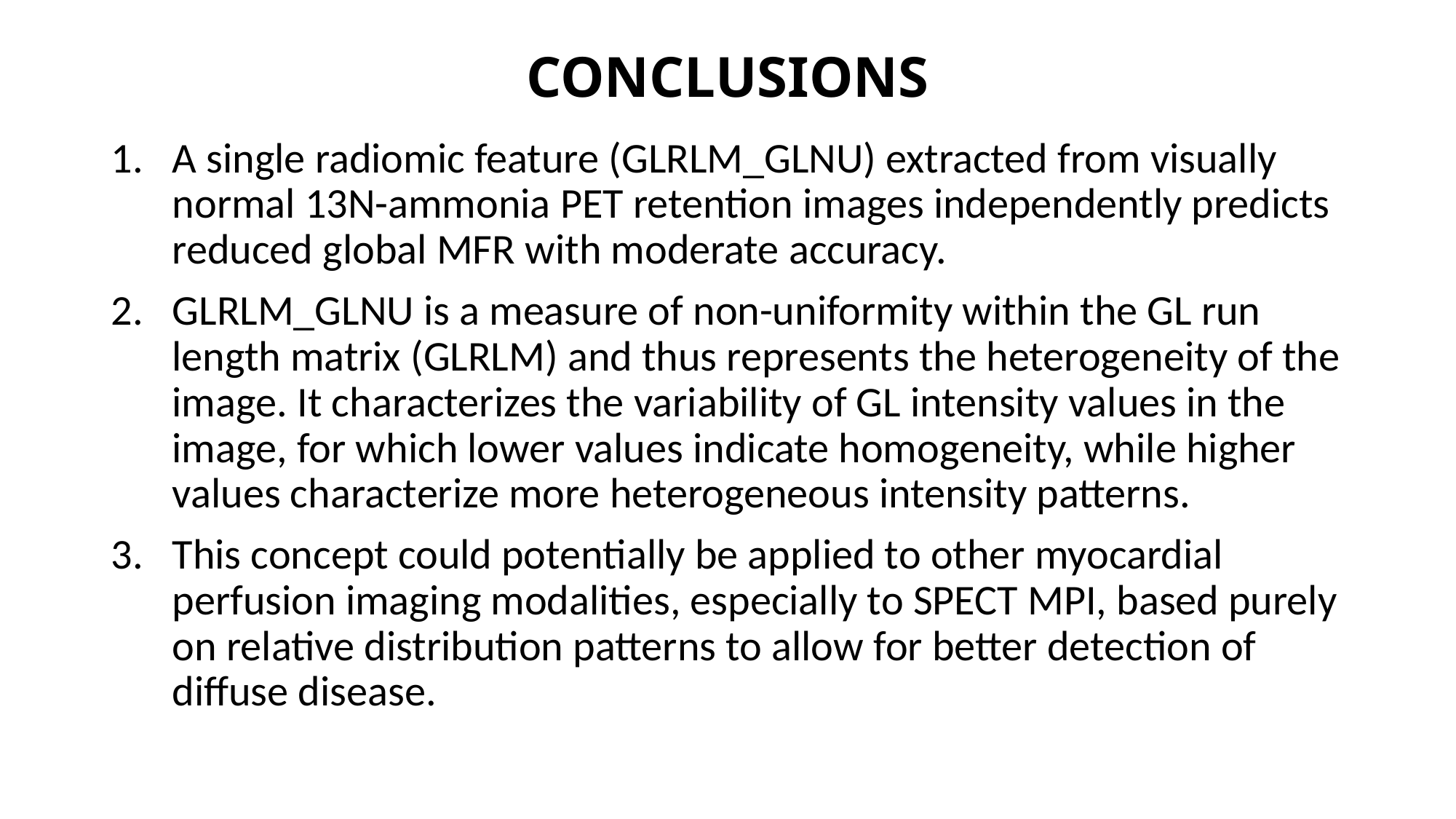

# CONCLUSIONS
A single radiomic feature (GLRLM_GLNU) extracted from visually normal 13N-ammonia PET retention images independently predicts reduced global MFR with moderate accuracy.
GLRLM_GLNU is a measure of non-uniformity within the GL run length matrix (GLRLM) and thus represents the heterogeneity of the image. It characterizes the variability of GL intensity values in the image, for which lower values indicate homogeneity, while higher values characterize more heterogeneous intensity patterns.
This concept could potentially be applied to other myocardial perfusion imaging modalities, especially to SPECT MPI, based purely on relative distribution patterns to allow for better detection of diffuse disease.
